# Pharmacological manipulation of nested oscillations in human iPSC-derived 2D neuronal networks

**DOI:** 10.1016/j.nbd.2026.107281

**Published:** 2026-01-24

**Authors:** Deborah Pré, Christian Cazares, Alexander T. Wooten, Haowen Zhou, Isabel Onofre, Ashley Neil, Todd Logan, Ruilong Hu, Jan H. Lui, Bradley Voytek, Anne G. Bang

**Affiliations:** a Conrad Prebys Center for Chemical Genomics, Sanford Burnham Prebys Medical Discovery Institute, 10901 North Torrey Pines Road, La Jolla, CA 92037, USA; b Department of Cognitive Science, University of California, San Diego, La Jolla, CA 92037, USA; c Halıcıoğlu Data Science Institute, University of California, San Diego, La Jolla, CA 92037, USA; d Kavli Institute for Brain and Mind, University of California, San Diego, La Jolla, CA 92037, USA; e BioMarin Pharmaceutical, 105 Digital Drive, Novato, CA 94949, USA

**Keywords:** Human induced pluripotent stem cell (hiPSC), Oscillations, Excitation/inhibition, Multi-electrode array, Cholinergic system, Potassium channels

## Abstract

Dynamically coupled neural networks are fundamental to human cognition and behavior and are disrupted in neurodevelopmental disorders. The formation and dissolution of functional networks is thought to be driven by synchronized oscillatory bursts across large populations of neurons. The mechanisms driving the emergence of these rhythms, known as oscillogenesis, are not well understood, particularly in the human brain. Using multi-electrode arrays, we investigated oscillogenesis in human induced pluripotent stem cell 2D neural cultures at different developmental stages and under pharmacological challenges. We found that cultures exhibited nested oscillations that were reduced by GABAA receptor blockade and emerged earlier when the proportion of GABAergic neurons was increased. Pharmacological manipulations of voltage-gated potassium channels and cholinergic receptors modulated the pattern of nested oscillations. These results reveal the capacity of these 2D cultures to model oscillogenesis, and underscore the need for their continued refinement, paving the way for linking systems-level neural networks to human cognition and disease.

## 1. Introduction

Neural oscillations are a characteristic and emergent feature in brain recordings. Across species, oscillatory neural activity facilitates the synchronization of neuronal assemblies and has been linked to fundamental cognitive and behavioral processes, including decision-making, learning, memory, attention and sensory integration (Buzsaki and Draguhn, 2004; Kucewicz et al., 2024; Palva and Palva, 2007; van Bree et al., 2025; Voytek and Knight, 2015). Due to their ubiquity in neural recordings, oscillations are hypothesized to serve as critical markers of underlying biological mechanisms that enable healthy self-organization of rhythmic neural activity (Fries, 2005; Gao et al., 2020; Singer, 1999). Substantial evidence for this comes from the developmental transitions observed as synaptic connections proliferate and inhibitory interneurons mature, during which sparse, ‘noise-like’ neural activity changes into synchronized network activity during early neurodevelopment, followed by the transition toward partial desynchronization upon further maturation (Chini et al., 2022; Khazipov and Luhmann, 2006; Wu et al., 2024). Furthermore, disruptions to the development of oscillatory neural activity patterns, referred to as oscillogenesis, are linked to many neurological and psychiatric conditions, such that oscillatory disruptions often precede the appearance of clinical symptoms (Chini and Hanganu-Opatz, 2021; Halje et al., 2019; Ippolito et al., 2022; Jafari et al., 2020; Pan et al., 2021; Weiss et al., 2023). But despite the prominence of oscillations as markers for human health and disease, the neurobiological underpinnings of their developmental origins remain elusive.

Advances in methodologies to produce neural tissue from human induced pluripotent stem cells (hiPSCs) (Takahashi et al., 2007) have led to unique opportunities to identify the molecular and neural circuit origins of oscillatory patterns, with unprecedented spatiotemporal resolution, during pre-birth stages that are otherwise difficult to study during fetal cortex development. These methods include relatively lengthy “directed-differentiation” protocols that recapitulate developmental processes from progenitors to differentiated neural cell types in both two-dimensional (2D) neural tissue and three-dimensional (3D) neural organoid formats, as well as very rapid 2D approaches that bypass progenitor stages via overexpression of transcription factors such as Neurogenin-2 (NGN2) (Hulme et al., 2022).

A key feature of hiPSC-derived neural tissue cultures is their ability to capture functional aspects of early human neural development, including oscillogenesis, as reflected by the onset of synchronized network spiking and the emergence of complex oscillatory dynamics observed in multi-electrode array (MEA) recordings of 3D neural organoids (Samarasinghe et al., 2021; Sharf et al., 2022; Trujillo et al., 2019) and 2D directed-differentiation neural tissue cultures (Salimpour et al., 2024). These models have also provided insights into the underlying cellular and molecular mechanisms of oscillogenesis, including an essential role of GABAergic signaling in the regulation of oscillatory activity. For instance, mature 3D hiPSC-derived neural organoids (6-months old) display oscillations nested within low-frequency network bursts, which have been found to critically depend on gamma-aminobutyric acid (GABA)ergic signaling (Trujillo et al., 2019). Complementing this finding, 2D hiPSC-derived neural tissue cultured for >6 months displays GABA-dependent phase-amplitude coupling between low (1–10 Hz) and high (20–200 Hz) frequencies, thought to be a marker for fundamental cognitive and behavioral processes in humans (Salimpour et al., 2024). Further work has shown that the cellular makeup and functional connectivity of 3D hipsc-derived neural organoid models can influence the emergence of gamma (30–100 Hz) oscillations and can serve as a therapeutic discovery platform for investigating the origins of epileptiform and atypical rhythmic activity in developmental conditions, including rett syndrome (Samarasinghe et al., 2021). Synchronized network spiking has also been reported in multiple studies of NGN2-induced 2D neural tissue cultures, with some referring to it as oscillatory activity (Rasanen et al., 2024), fragmented network bursts (Doorn et al., 2024), high frequency bursts (Van Hugte et al., 2023), or reverberating super bursts (Pradeepan et al., 2024). Moreover, gabaergic neurons and/or response to GABAA receptor (GABAAR) blockade were described as absent in most of these studies (Pradeepan et al., 2024; Rasanen et al., 2024; van Hugte et al., 2023), suggesting that GABA is not necessary for synchronized network spiking in NGN2-induced neurons.

However, despite their promise, previous studies on the mechanisms of oscillogenesis in hiPSC-derived models have critical limitations that hinder their translational potential. Extensive work has focused on 3D neural organoids cultured for prolonged periods (>6 months), which, while physiologically relevant to human neural tissue, pose significant challenges for drug discovery applications due to their high cost, variability, and limited throughput (Chiaradia and Lancaster, 2020). And while 2D neuronal tissue cultures provide a more economical and scalable early-stage screening platform, their potential for modeling complex oscillatory dynamics has not been adequately characterized in a way that considers potential confounds of non-oscillatory activity, which has impeded their utility for accurately modeling the developmental trajectory of aberrant oscillatory brain activity in neurological and psychiatric disorders. Previous work has also not systematically investigated how the proportions of glutamatergic and GABAergic neurons influence oscillogenesis, which specific receptor systems and channels modulate these oscillatory patterns, and how they differ across differentiation protocols. Furthermore, hiPSC-derived models have displayed aperiodic (broadband) signal characteristics in MEA recordings in parallel with oscillatory activity (Trujillo et al., 2019), yet the origins of this “noise-like” aspect of neural signals remain largely unexplored, despite the growing literature linking this feature to excitatory-inhibitory (E/I) balance in healthy and perturbed cortical networks (Donoghue et al., 2020; Gao et al., 2017; McKeon et al., 2024; Ostlund et al., 2022).

Here, we systematically investigated mechanisms underlying human neural network oscillogenesis in 2D hiPSC-derived neuronal networks, using MEA recordings to characterize the development of synchronized network spiking. In iCell GlutaNeurons (iGluta, FujiFilm Cellular Dynamics Inc. (FFCDI)), a neuronal population generated via directed differentiation that is primarily comprised of glutamatergic neurons with a minor GABAergic population, we documented the emergence of nested oscillations in delta (1–4 Hz), theta (4–8 Hz), and alpha (8–12 Hz) frequency ranges, and analyzed their oscillatory properties and aperiodic signal components. These nested oscillations depend on GABAergic signaling, as they were reduced by GABAAR blockade, and increasing the proportion of GABAergic neurons accelerated their onset and modified their pattern. Pharmacological studies revealed distinct roles for voltage-gated potassium (Kv) channels: broad-spectrum Kv channel blockade enhanced oscillation duration and power in iGluta cultures, whereas selective Kv7.2/7.3 inhibition had opposite effects. We also observed complex temporal responses to cholinergic receptor agonists and antagonists, with significant impacts on oscillatory parameters and aperiodic signal characteristics. In contrast, NGN2-patterned induced neurons (NGN2-piNs) (Nehme et al., 2018) exhibited only rudimentary nested oscillations that were unaffected by GABAergic blockade, despite the presence of GABAergic neurons, and showed increased oscillation duration in response to Kv7.2/7.3 inhibition. Together these results underscore potential differences in oscillogenesis in neurons generated via directed differentiation versus NGN2-overexpression. Overall, our findings support the potential of 2D hiPSC-derived neuronal cultures as economical and scalable platforms for drug screening and modeling network oscillatory dysfunction relevant to human cognition in health and disease.

## 2. Results

### 2.1. 2D cultures of hiPSC-derived neurons generated via directed differentiation develop nested oscillations

In this study, we investigated the development of coordinated neuronal activity in 2D cultures of hiPSC-derived neurons (iCell GlutaNeurons, iGluta, (FFCDI)) co-cultured with either iCell Astrocytes (iAstro, FFCDI) or with primary human astrocytes (PHA, ScienceCell) at an 8:1 ratio. PHA and iAstro are highly enriched populations of astrocytes based on expression of GFAP and CD44, which do not exhibit neuronal marker expression (Fig. S1A and B), as shown in comparison to iGluta-alone (Fig. S1C), and in iGluta co-cultures (Fig. S1D and E). We observed that network activity of iGluta co-cultured with iAstro (added at the time of plating) versus PHA (added after 7 days) was indistinguishable in our assays when comparing weighted mean firing rate (WMFR) and number of network bursts (NB) (Fig. S1F and G). For brevity, we refer to iGluta and astrocyte co-cultures simply as “iGluta”, and indicate astrocyte type, iAstro or PHA, in the figure legends. iGluta neurons are generated using directed differentiation based on a modified version of a dual-SMAD inhibition (DSI) protocol (Chambers et al., 2009) (personal communication, FFCDI). Based on manufacturer specifications, iGluta are ~70–90% glutamatergic, depending upon the lot. Bulk RNAseq analyses showed that they express the lower layer cortical marker TBR1, and GABAergic and glutamatergic markers (Fig. S2A). Immunofluorescence (IF) confirmed the presence of GABA-expressing neurons (Fig. S2B), as well as TBR1-positive neurons (Fig. S2C).

**Fig. 1.**
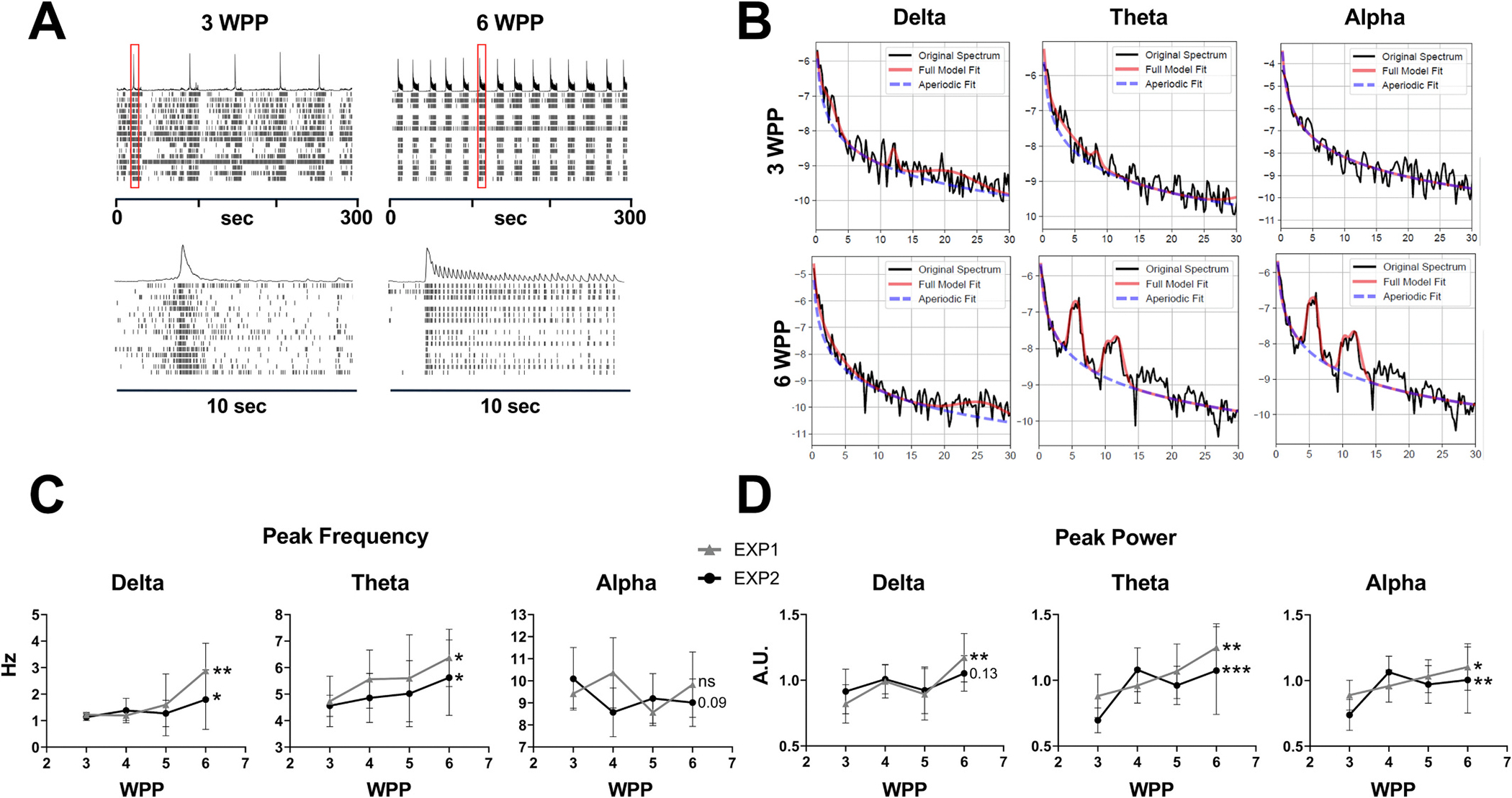
Development of nested oscillatory activity in iGluta cultures over time. (A) Representative raster plots from MEA recordings showing organized network activity of iGluta/iAstro co-cultures at 3 and 6 WPP. Red boxes indicate 10 s interval insets shown below each panel. Each row of the raster plot shows a recording from a single electrode, out of 16 total, and the histogram at the top shows the sum of well-wide spikes at the indicated time in seconds (sec). (B) Power spectral density and *specparam* analysis illustrating modeled aperiodic fit for spectral power peak detection within the delta (1–4 Hz), theta (4–8 Hz), and alpha (8–13 Hz) frequency ranges at 3 WPP (top row) and 6 WPP (bottom row) in iGluta/iAstro co-cultures. (C and D) Temporal progression of oscillatory features in two independent experiments for iGluta/iAstro co-cultures (EXP1 and EXP2): (C) Peak frequency and (D) peak aperiodic-corrected power of nested oscillations. *n* = 6 wells for EXP1 and *n* = 15 wells for EXP2. All data are shown as mean ± SD. *p*-values were calculated using a repeated measure one-way ANOVA and indicate significance of a linear trend. *p* values are indicated as: *0.05 > *p* ≥ 0.01; **0.01 > *p* ≥ 0.001; ***0.001 > *p* ≥ 0.0001; ****p < 0.0001. (For interpretation of the references to colour in this figure legend, the reader is referred to the web version of this article.)

**Fig. 2.**
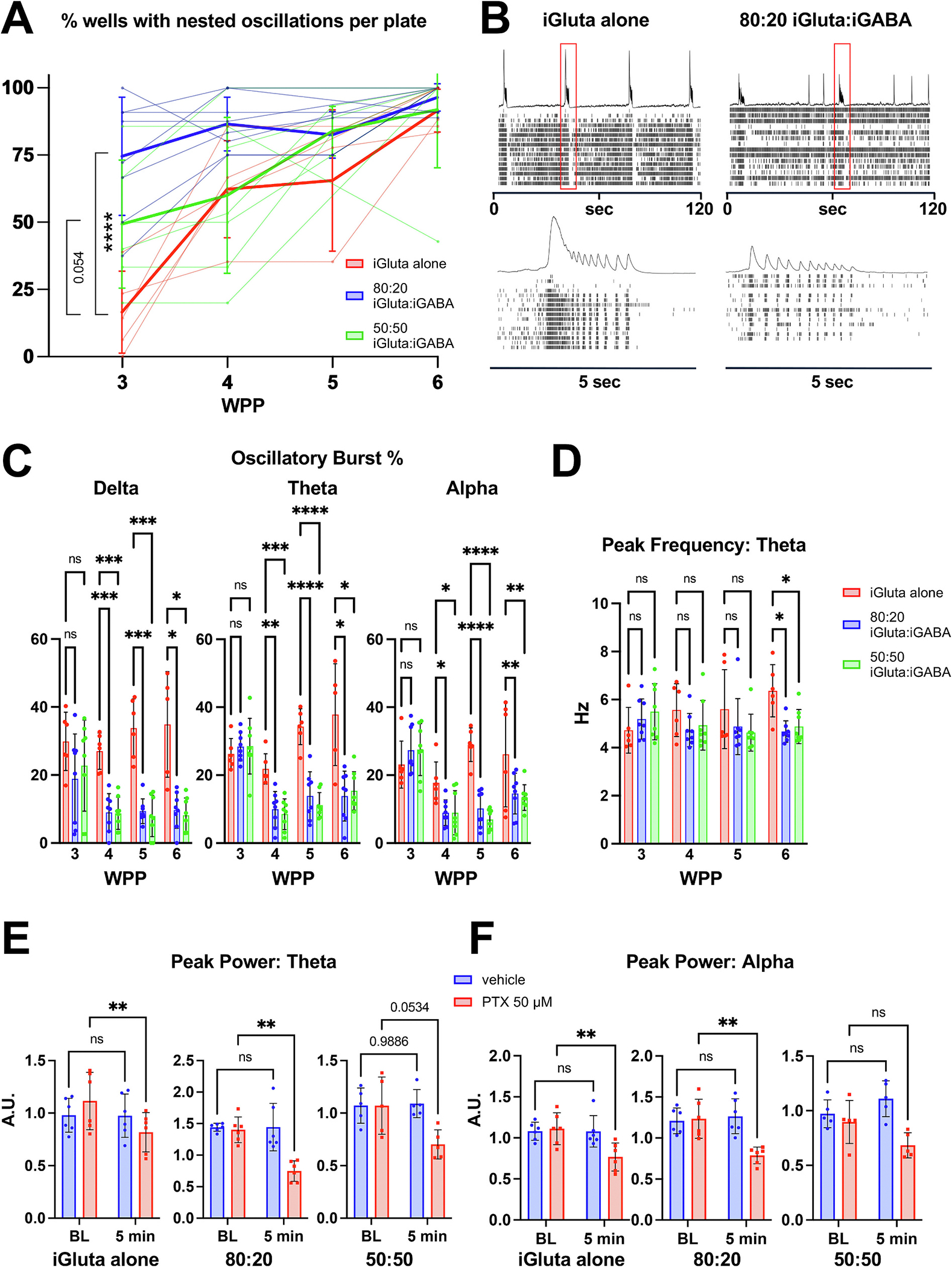
Contribution of the inhibitory system to nested oscillations. (A) Percentage of wells exhibiting nested oscillations over 3–6 weeks in cultures for iGluta-alone (red), 80:20 iGluta:iGABA (blue), or 50:50 iGluta:iGABA (green). Each data point represents an individual plate; paler lines show jittered trajectories for individual plates, and darker lines indicate the group mean ± SD across plates. Relative to iGluta-alone cultures, 80:20 iGluta:iGABA mixed networks exhibited substantially higher overall odds of oscillation (*p* = 4.5 × 10^−6^). 50:50 iGluta:iGABA networks showed a similar but weaker trend toward higher overall nested oscillation levels (*p* = 0.054). Statistical analysis was performed using a generalized estimating equation (GEE). *n* = 5 plates with a total of 71 wells and a range of 6 to 18 wells per plate for iGluta-alone; *n* = 8 plates with a total of 87 wells and a range of 5 to 18 wells per plate for 80:20 iGluta:iGABA; *n* = 7 plates with a total of 49 wells and a range of 3 to 15 wells per plate for 50:50 iGluta:iGABA. See Table S1 for full model parameters and odds ratios. (B) Representative raster plots from MEA recordings of iGluta-alone and 80:20 iGluta:iGABA co-cultures at 6 WPP. Red boxes in the top panels indicate 5 s interval insets shown in the bottom panels. (C) Percentage of oscillatory bursts in delta, theta, and alpha frequency ranges at 3, 4, 5 and 6 WPP for iGluta, 80:20 iGluta:iGABA, and 50:50 iGluta:iGABA co-cultures. (D) Theta peak frequency at 3, 4, 5 and 6 WPP for iGluta-alone, 80:20 iGluta:iGABA, and 50:50 iGluta:iGABA co-cultures. For (C) and (D): *n* = 6 wells for iGluta-alone, n = 8 wells for 80:20 iGluta:iGABA, and n = 8 wells for 50:50 iGluta:iGABA co-cultures. Data shown are representative of three independent experiments. (E and F) Analyses of responses to 50 μM PTX treatment for the three conditions (iGluta-alone, 80:20 iGluta:iGABA, and 50:50 iGluta:iGABA co-cultures), showing theta (E) and alpha (F) aperiodic-corrected peak power at baseline and 5 min post-treatment at 5 WPP. For (E) and (F) both vehicle and PTX conditions: n = 6 wells for iGluta-alone and 80:20 iGluta:iGABA, and n = 5 wells for 50:50 iGluta:iGABA co-cultures. Data shown are representative of two independent experiments. All data are shown as mean ± SD. *p*-values were calculated using two-way ANOVA with Dunnett’s multiple comparison test (C and D), and two-way ANOVA with Sidak’s multiple comparison test (E and F) and are indicated as: *0.05 > *p* ≥ 0.01; **0.01 > *p* ≥ 0.001; ***0.001 > *p* ≥ 0.0001; ****p < 0.0001. Data shown are representative of at least 2 experiments. (For interpretation of the references to colour in this figure legend, the reader is referred to the web version of this article.)

We used 48-well MEA plates (Axion BioSystems) to measure and pharmacologically manipulate neural activity across multiple, independent neuronal networks. As early as two weeks post-plating (WPP) of iGluta cultures on MEAs, we observed low-frequency (<1 Hz) bursts of synchronized network spiking that become more robust by 3 WPP (Fig. 1A), in line with prior reports of emergent network activity in these cultures (Tukker et al., 2020; Tukker et al., 2018). Between ~3–4 WPP, network activity began to display higher frequency synchronized network spiking resembling oscillations nested within lower frequency network bursts, a pattern that we observed increasingly from 4 to 6 WPP in terms of number of wells that exhibited this behavior (Fig. 1A, see also Fig. 2A). To characterize this network activity, we used a convolution process to transform discrete spike events into a continuous network signal that approximated the underlying temporal dynamics of the neuronal population activity (Ness et al., 2025) (Fig. S3A and B). This approach allowed us to quantify features of network activity using standard spectral analysis techniques typically applied to local field potential recordings. We then isolated delta (1–4 Hz), theta (4–8 Hz), and alpha (8–13 Hz) frequency components using bandpass filtering and detected oscillatory bursts via amplitude envelope thresholding. For each detected burst, we calculated the power spectral density of the original continuous signal and used *specparam* (Donoghue et al., 2020) to quantify aperiodic-corrected oscillatory power, allowing us to estimate the representation of specific frequency bands during network activity while taking into account non-oscillatory background activity (Fig. S3C–E).

Fig. 1B shows representative power spectra with model fits from individual detected bursts within each frequency band at 3- and 6-WPP. We tracked the temporal development of these oscillatory features across two independent experiments, in which iGluta neurons were plated on MEAs on different dates (Fig. 1C and D). Over time, the peak frequency increased in the delta and theta ranges, but not in the alpha range (Fig. 1C). Moreover, an increase in spectral power at the peak frequency in all frequency ranges was observed (only trending in the delta frequency for one of the two experiments) (Fig. 1D). Our analytical approach revealed previously uncharacterized complexity in the emergence of oscillatory activity in 2D neural cultures, demonstrating that these simplified in vitro systems recapitulate key temporal features of early cortical network development observed in vivo.

We also tracked the progression of organized activity and the emergence of nested oscillations in hiPSC-derived cortical neurons generated in-house using a directed-differentiation protocol on a line harboring a heterozygous DISC1 mutation (Wen et al., 2014). Cortical neurons were differentiated from the neural progenitor cell (NPC) stage for 4 weeks to generate a population enriched for MAP2-positive neurons expressing the lower layer cortical marker TBR1 (Fig. S4A), as well as a smaller contribution of GABA-positive GABAergic neurons (Fig. S4B). Neurons were dissociated and cultured alone, or in co-cultures with PHA on MEAs (Fig. S4C). At approximately 3 WPP we observed the emergence of nested oscillations (Fig. S4D). Moreover, the addition of PHA induced a significant change in the oscillatory pattern with increasing peak frequency in the delta range (Fig. S4E) and mean aperiodic-corrected peak power in the theta and alpha range (Fig. S4F), recapitulating the critical role for astrocytes in supporting neural oscillations reported by others (Bellot-Saez et al., 2018; Rasanen et al., 2024). Oscillations in the delta, theta, and alpha frequency ranges were detected in one additional batch of the same in-house generated cortical neuronal line (Fig. S4G).

Together, these findings demonstrate that iGluta and in-house hiPSC-derived cortical 2D neuronal cultures generated via directed differentiation using DSI protocols can exhibit oscillatory activity that evolves over time; however, for in-house generated neurons, culture conditions required to consistently promote the development of nested oscillatory activity across different lines and batches require further optimization.

### 2.2. Contribution of the inhibitory system to nested oscillations

Given the role of GABAergic neurons in modulating oscillatory brain activity (Warm et al., 2021; Wu et al., 2024), we next evaluated inhibitory activity in iGluta. We first examined the effect of the GABAAR blocker picrotoxin (PTX) on slow NBs (<1 Hz) at 3 WPP, prior to the appearance of nested oscillations. Consistent with the presence of GABAergic neurons based on bulk RNAseq and IF (Fig. S2A and B), and as reported by others (Robin et al., 2020; Tukker et al., 2020), iGluta exhibited a robust response to PTX (Fig. S5A), characterized by increased percentage of spikes in bursts (burst %), a reduction in the inter-spike interval (ISI) within NBs, and increased synchrony index, a measure of the cross-correlation between spiking on all pairwise combinations of the electrodes (Halliday et al., 2006) (Fig. S5B). The effect of PTX on iGluta neurons was consistent across five experiments, and was confirmed using two additional GABAAR blockers, bicuculline and gabazine (data not shown).

To test whether altering E/I balance modulates network bursting, we co-cultured iGluta neurons with hiPSC-derived iGABA neurons (FFCDI), a population that, as reported by others (Boulting et al., 2021), we observed to be highly enriched for GABAergic neurons (Fig. S2D). Co-cultures at ~80:20 or 50:50 iGluta:iGABA ratios exhibited robust responses to PTX (Fig. S5B). The 80:20 cultures showed PTX responses comparable to iGluta-alone, suggesting both reached a minimal inhibitory threshold sufficient for PTX sensitivity. In comparison, 50:50 cultures showed attenuated PTX effects, consistent with excessive inhibition and a disrupted E/I equilibrium. Network responses to the GABA reuptake inhibitor tiagabine further distinguished these conditions: while iGluta/iGABA co-cultures displayed decreased synchrony and reduced network burst organization, iGluta-alone cultures were unaffected (Fig. S5C). These data suggest that tiagabine requires a critical level of inhibitory input to alter network behavior. Together, these findings highlight the importance of appropriate tuning of inhibitory neuron ratios, and that both deficient and excessive inhibition can change network organization in line with the narrow E/I operating range observed in developing cortical circuits (Chen et al., 2022a; Froemke, 2015).

Given the long-established role of E/I balance in healthy neurodevelopment, we next examined the impact of adding inhibitory neurons on the development of nested oscillations. For this analysis we tracked the percentage of wells exhibiting nested oscillations over 3 to 6 WPP of iGluta-alone, 80:20 iGluta:iGABA, or 50:50 iGluta:iGABA across 5, 8, and 7 independent plates, respectively. First, we identified wells which exhibited network bursting at 6 WPP. The emergence of the nested oscillations was then tracked in these wells at 3, 4, 5, and 6 WPP. Statistical analysis of these data was performed using a generalized estimating equation (GEE). Across all culture conditions, we observed the fraction of oscillating wells increased significantly between 3 and 6 WPP (*p* = 2.3 × 10^−8^). Relative to iGluta-alone, 80:20 iGluta:iGABA cultures exhibited substantially higher overall odds of oscillation (*p* = 4.5 × 10^−6^) and a significant time × condition interaction (*p* = 0.0002) confirmed that nested oscillations in these networks emerged earlier and reached plateau sooner than in iGluta-alone cultures, suggesting a modest increase in the inhibitory component accelerates the emergence of nested oscillations. 50:50 iGluta:iGABA networks showed a similar but weaker trend toward higher overall nested oscillation levels (*p* = 0.054), but their time × condition interaction was not significant (*p* = 0.83), indicating that while 50:50 iGluta:iGABA cultures tended to exhibit nested oscillations slightly more overall than iGluta-alone cultures, their temporal trajectory paralleled that of iGluta-alone cultures (Fig. 2A, see Table S1 for full model parameters). In addition, we observed that iGABA altered the oscillatory pattern, with iGluta/iGABA co-cultures exhibiting a mix of NBs with and without nested oscillations, compared to iGluta-alone cultures (Fig. 2B). We quantified this phenomenon across multiple time points from 3 to 6 WPP and found that, while there were no significant differences between iGluta-alone and iGluta/iGABA co-cultures at 3 WPP, at 4 to 6 WPP the iGluta-alone cultures exhibited a higher percentage of oscillatory NBs across all three frequency bands (Fig. 2C). In contrast, peak frequencies were similar across culture conditions from 3 to 6 WPP, with only iGluta-alone cultures exhibiting an increased peak frequency in the theta band at 6 WPP (Fig. 2D) and no change for the delta and alpha bands (data not shown), suggesting that while the pattern of oscillatory bursts differs between these culture conditions, the peak frequencies are mostly similar.

Our bulk RNAseq analyses of iGluta neurons (Fig. S2A) and published analyses of iGABA neurons (Boulting et al., 2021) show that both of these cell populations exhibit somatostatin (SST) expression, but only very low levels of parvalbumin (PVALB, PV), aligning with the temporal maturation patterns of GABAergic neuronal subtypes, wherein SST+ neurons mature earlier than PV+ neurons (Miyamae et al., 2017; Tuncdemir et al., 2016; Warm et al., 2021). The level of SST expression observed in iGluta (Fig. S2A) and iGABA (Boulting et al., 2021) neuronal populations is consistent with the possibility that SST+ interneurons contribute to regulating oscillatory behavior in this system. These results support the idea that differing ratios of GABAergic and glutamatergic input can be used as a tool to explore development of nested oscillations in 2D cultures, a format that, compared with brain organoids, allows for more control over composition of the cell population and provides a platform to test predominant hypotheses regarding E/I balance in neurodevelopmental conditions (Marx, 2018).

To confirm whether inhibitory input modulates nested oscillations, we applied PTX. We observed that PTX treatment reduced nested oscillations (Fig. S5D) and significantly decreased the aperiodic-corrected peak power of the nested oscillations in the theta and alpha frequency ranges (Fig. 2E and F). This effect was weaker in the 50:50 iGluta:iGABA co-cultures, where the reduction in the aperiodic-corrected peak power in both the theta and alpha ranges trends down but does not reach significance. As discussed above, a possible interpretation of this result is that response of the 50:50 condition to PTX reflects an E/I imbalance in the network that is tipped too far toward inhibition.

These findings align with previous studies demonstrating that GABA-mediated inhibition modulates neural oscillations in hiPSC-derived brain organoids (Samarasinghe et al., 2021; Sharf et al., 2022; Trujillo et al., 2019) and 2D networks generated with directed differentiation (Salimpour et al., 2024), positioning 2D cultures as complementary models to 3D brain organoids to study the development of nested oscillations in disease contexts, as well as their response to drug treatments.

### 2.3. Voltage-gated potassium channels modulate nested oscillations

To assess the translational potential for pharmacologically manipulating oscillations in 2D hiPSC neuronal cultures, we examined their modulation by antagonists of Kv channels for which pathogenic variants have been implicated in neurological disorders like epilepsy and neurodevelopmental syndromes (Springer et al., 2021).

**Fig. 3.**
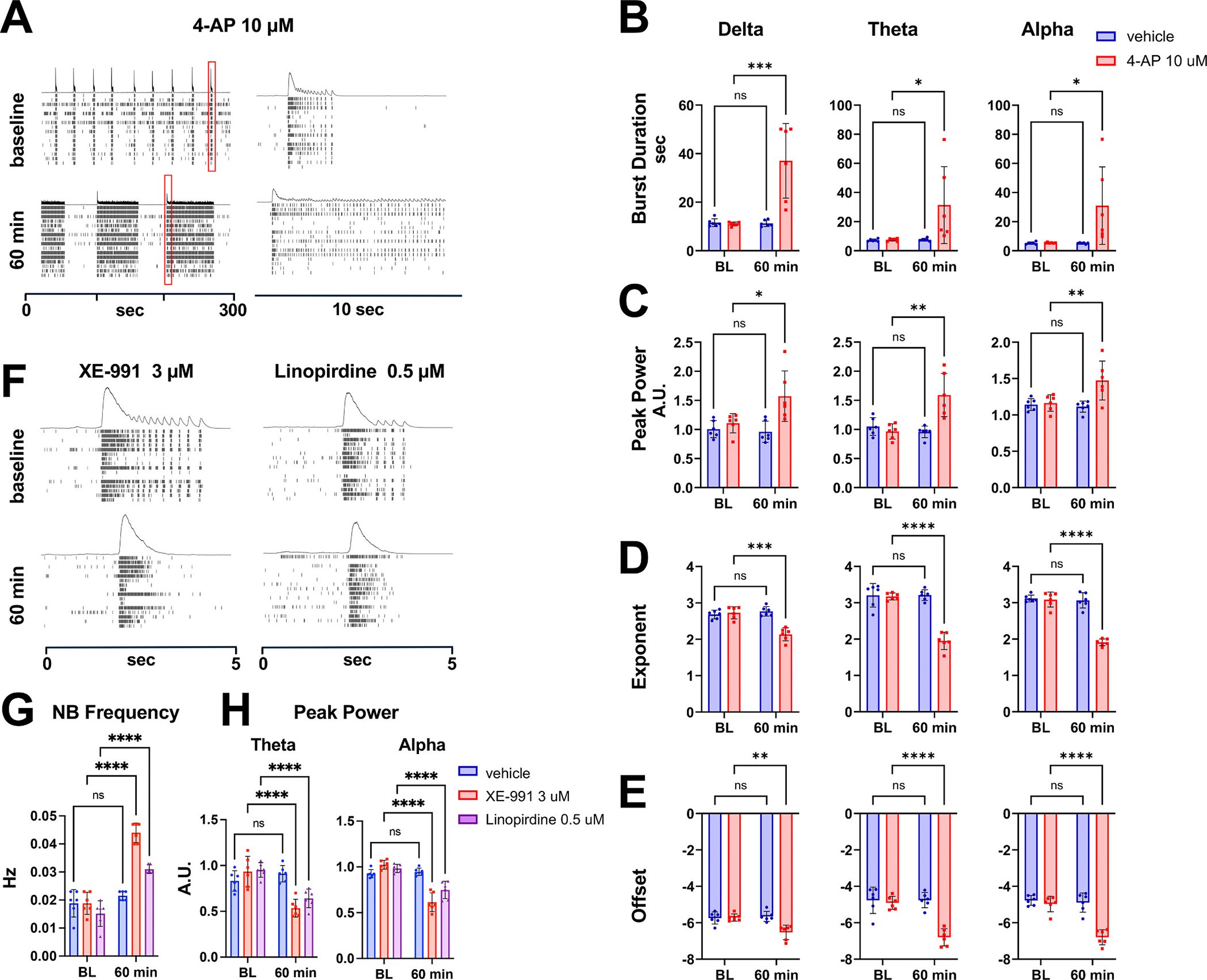
Voltage-gated potassium channel contribution to oscillatory behavior in iGluta cultures. (A) Representative raster plots from MEA recordings of iGluta/PHA co-cultures treated with 10 μM 4-AP for 60 min. Red boxes indicate 10 s interval insets shown to the right of each panel. (B-E) Analysis of oscillatory bursts in iGluta cultures at 4 WPP, showing burst duration (B), aperiodic-corrected peak power (C), exponent (D) and offset (E) in the delta (1–4 Hz), theta (4–8 Hz), and alpha (8–13 Hz) frequency ranges at baseline and 60 min post-treatment with 4-AP (10 μM) for the experiment shown in panel A. n = 6 wells per condition. Data shown are representative of two independent experiments. (F) Representative raster plots of iGluta/PHA co-cultures at baseline and 60 min post-treatment with XE-991 (3 μM) or linopirdine (0.5 μM). (G) Number of network bursts at baseline and 60 min for XE-991-treated or linopirdine-treated iGluta cultures at 4 WPP, for the experiment shown in panel F. (H) Oscillatory burst aperiodic-corrected peak power in iGluta cultures at baseline and after 60 min of treatment with XE-991 (3 μM) or linopirdine (0.5 μM), for the experiment shown in panel F. (F-H): n = 6 wells per condition. Data shown for F-H are representative of 2 independent experiments for linopirdine and 2 independent experiments for XE-991. All data are shown as mean ± SD. *p*-values were calculated using two-way ANOVA with Sidak’s multiple comparison test and are indicated as: *0.05 > *p* ≥ 0.01; **0.01 > *p* ≥ 0.001; ***0.001 > *p* ≥ 0.0001; ****p < 0.0001. Data shown are representative of at least 2 experiments. (For interpretation of the references to colour in this figure legend, the reader is referred to the web version of this article.)

In oscillating iGluta cultures treated with 4-aminopyridine (4-AP), a broad spectrum Kv channel blocker, we observed a significant increase in the duration and aperiodic-corrected peak power of nested oscillations in all frequency bands (Fig. 3A–C), likely due to a delayed hyperpolarization phase and subsequent longer bursts. We also observed a reduction in the aperiodic activity measures (exponent and offset) of the power spectrum for all frequency bands (Fig. 3D and E). These measures, derived from our *specparam* analysis, characterize the background 1/f-like component of the neural signal, with the offset reflecting the overall power and the exponent describing the slope of the power decay across frequencies (Donoghue et al., 2020). The reduction in these measures following 4-AP treatment suggests that Kv channel blockade alters not only the oscillatory components but also the underlying aperiodic neural activity, potentially reflecting broader changes in network excitability beyond any one single frequency band.

Next, we evaluated the effects of two specific Kv7.2/7.3 channel blockers, XE-991 and linopirdine (Brown and Passmore, 2009), on nested oscillations in iGluta cultures at 4 WPP on MEAs (Fig. 3F). Both treatments increased the NB frequency but reduced the aperiodic-corrected peak power of nested oscillations in the theta and alpha frequency ranges (Fig. 3G and H). The observed effect of XE-991 was confirmed at a later developmental stage (Fig. S6A and B). Consistent with earlier findings, XE-991 treatment increased network burst (NB) frequency and reduced peak power in the alpha frequency band, while producing only a modest, non-significant decrease in the theta frequency band at 6 WPP (Fig. S6B).

These findings underscore the potentially distinct roles of Kv channel subtypes in modulating network dynamics, with some subtypes promoting and others constraining the emergence of oscillatory activity in directed-differentiation 2D hiPSC-derived networks.

### 2.4. Cholinergic agonists and antagonists modulate nested oscillations

**Fig. 4.**
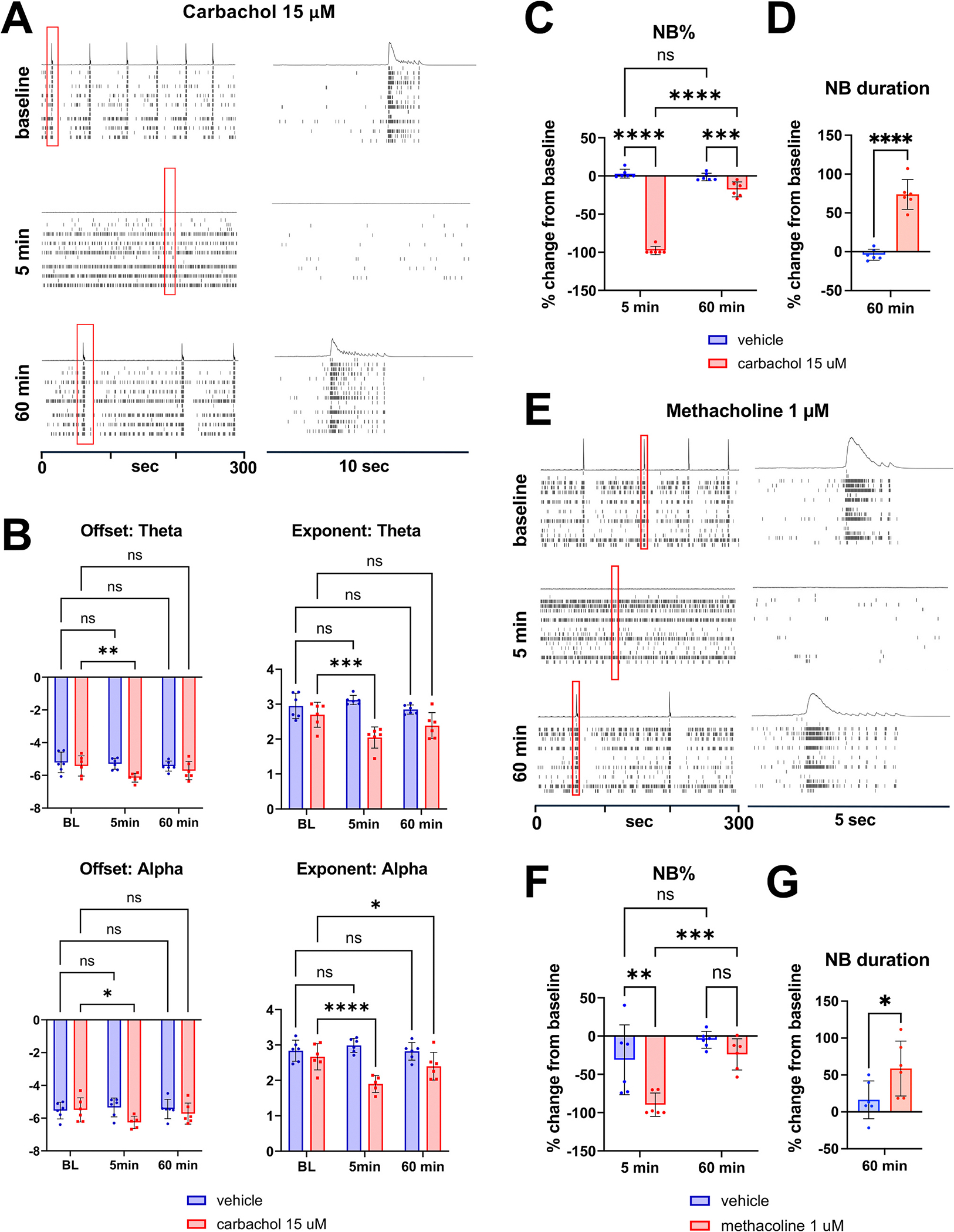
Response of nested oscillations in iGluta cultures to carbachol and methacholine. (A) Representative raster plots from MEA recordings of iGluta/PHA co-cultures treated with 15 μM carbachol at 4 WPP. Raster plots display 300-s recordings at baseline, 5 and 60-min post-treatment. Red boxes highlight 10 s interval insets shown in the right panels. (B) Analysis of offset and exponent in the theta and alpha frequency bands at baseline and after 5 and 60 min of 15 μM carbachol treatment of iGluta/PHA co-cultures. (C) Quantification of NB% for vehicle and 15 μM carbachol-treated iGluta/PHA co-cultures at 5 min and 60 min post treatment expressed as % change from baseline. (D) Quantification of NB duration for vehicle and 15 μM carbachol-treated iGluta/PHA co-cultures at 60 min after treatment expressed as % change from baseline. (A-D) Data shown are representative of two independent experiments. (E) Representative raster plots of MEA recordings from iGluta/PHA co-cultures treated with 1 μM methacholine at baseline and at 5 and 60 min post treatment at 4 WPP. Red boxes highlight 5 s interval insets shown in the right panels. (F) Quantification of NB% for vehicle and 1 μM methacholine-treated iGluta/PHA co-cultures at 5 and 60 min post treatment expressed as % change from baseline. (G) Quantification of NB duration for vehicle and 1 μM methacholine-treated iGluta/PHA co-cultures at 60 min post treatment expressed as % change from baseline. n = 6 wells for all vehicle and drug treated samples. (E-G) Data in these panels are from a single experiment; the effect was reproduced using another muscarinic ACh receptor (mAChR) agonist, bethanechol (see Fig. S6C–E). All data are shown as mean ± SD. *p*-values were calculated using: two-way ANOVA with Tukey’s multiple comparison test (B); two-way ANOVA with Fisher LSD test (C, F); and unpaired *t*-test (D, G) and are indicated as: *0.05 > *p* ≥ 0.01; **0.01 > *p* ≥ 0.001; ***0.001 > *p* ≥ 0.0001; ****p < 0.0001. (For interpretation of the references to colour in this figure legend, the reader is referred to the web version of this article.)

Cholinergic neuromodulation plays a pivotal role in regulating oscillatory activity in both humans and rodent models (Buzsaki and Draguhn, 2004; Hanganu et al., 2007; Kilb and Luhmann, 2003; Weiss et al., 2023; Wu et al., 2024). To explore cholinergic modulation of iGluta cultures exhibiting oscillatory activity, we treated them with carbachol, an acetylcholinesterase resistant, non-selective cholinergic agonist that activates both muscarinic and nicotinic acetylcholine receptors (mAChRs and nAChRs). After 5 min of carbachol treatment, we observed a strong disruption in slow network bursting activity and significant reduction of offset and exponent in the theta and alpha frequency bands (Fig. 4A and B) and a strong reduction in network organization, measured as NB% at 5 min (Fig. 4C). These results are consistent with previous studies showing that carbachol induces a shift from synchronized network spiking to asynchronous single-spike activity (Colombi et al., 2021; Tateno et al., 2005). Notably, this initial effect reverted while still in the presence of drug, with slow network bursting and nested oscillations reappearing after 60 min of treatment (Fig. 4A and C), when an increase in NB duration was also observed (Fig. 4D). Similar results were obtained with 1 mM treatment of acetylcholine (ACh) (data not shown).

Our transcriptomic analyses revealed co-expression of mAChRs and nAChRs (Fig. S2A). To explore the contribution of these receptors to oscillogenesis, we used a pharmacological approach to selectively target them. Neither nicotine nor lobeline, two nAChR agonists, induced significant changes in oscillatory activity (data not shown). However, treatment with 1 μM methacholine or 100 μM bethanechol, two mAChR agonists, showed similar effects to carbachol treatment, with a temporary disruption in network organization that reverts after 60 min in the presence of the drug (Fig. 4 E and F for methacholine and Fig. S6C and D for bethanechol) when an increased NB duration is also observed (Fig. 4G for methacholine and S6E for bethanechol). These results indicate that modulation of the duration of nested oscillations by carbachol and ACh in iGluta is primarily mediated by mAChRs, most likely M2 and M3 subtypes based on their expression levels in bulk RNA-seq analyses (Fig. S2A).

**Fig. 5.**
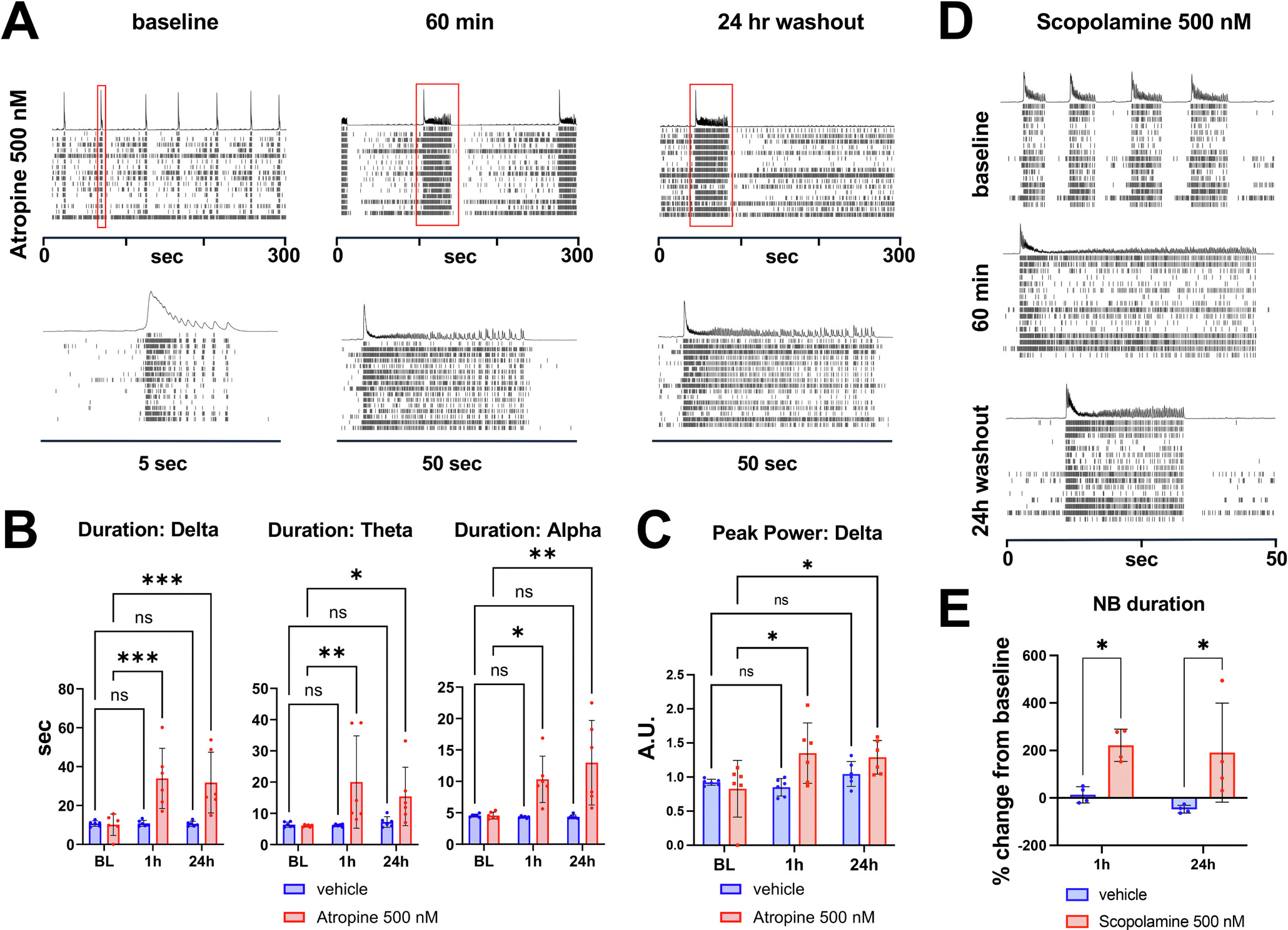
Response of nested oscillations in iGluta cultures to atropine and scopolamine. (A) Representative raster plots from MEA recordings of iGluta/PHA co-cultures treated with atropine (500 nM) at 3 WPP, showing 300-s recordings at baseline, 60 min post-treatment, and 24 h post drug washout. (B) Analysis of oscillatory burst duration in iGluta cultures at 4 WPP, 1 h post treatment with atropine (0.5 μM) and 24 h post-washout, as shown in panel A. The duration is shown for delta (1–4 Hz), theta (4–8 Hz), and alpha (8–13 Hz) frequency ranges. (C) Analysis of aperiodic-corrected peak power in the delta (1–4 Hz) frequency range at baseline and at 1 h and 24 h post vehicle or 0.5 μM atropine treatment. n = 6 wells per condition. Data shown are representative of three independent experiments. (D) Representative raster plots of MEA recordings from iGluta/iAstro cultures treated with 500 nM scopolamine at baseline, 60 min post-treatment, and 24 h after drug washout at 6 WPP. (E) Network burst duration at 1 h after vehicle or scopolamine treatment and 24 h post drug washout calculated as % change from baseline for the experiment shown in panel D. *n* = 4 wells for vehicle and scopolamine-treated samples. Data are representative of two independent experiments. All data are shown as mean ± SD. *p*-values were calculated using two-way ANOVA with Tukey’s multiple comparison test (B and C) and Sidak’s multiple comparison test (E) and are indicated as: *0.05 > *p* ≥ 0.01; **0.01 > *p* ≥ 0.001; ***0.001 > *p* ≥ 0.0001; ****p < 0.0001. Data shown are representative of at least 2 experiments.

Interestingly, treatment with atropine, a naturally occurring mAChR antagonist, resulted in a significant increase in the duration of nested oscillations in all three frequency bands at 60 min, persisting at 24 h after washout of the drug (Fig. 5A and B), suggesting effects on neuroplasticity. Moreover, we observed an increase in aperiodic-corrected peak power in the delta band at 1 h (Fig. 5C). A comparable increase in duration of oscillatory NBs at 1 h that persisted 24 h after washout was also observed after treatment with scopolamine (Fig. 5D and E), another mAChR antagonist similar in structure to atropine, which is under investigation as a fast-acting antidepressant (Anacker, 2018; Navarria et al., 2015). However, interpretation of these results is confounded by the apparent lack of cholinergic neurons in the iGluta cell population as ChAT-positive neurons were not detected by IF (data not shown), and bulk RNAseq analysis revealed only very low levels of ChAT expression (Fig. S2A). In addition, despite the presence of acetylcholinesterase (AChE) based on RNAseq data (Fig. S2A), treatment with the AChE inhibitors donepezil and rivastigmine had no apparent effect on nested oscillations or network activity (data not shown). Thus, the mechanism of action of atropine and scopolamine remains unclear (see Discussion).

### 2.5. NGN2-induced neuronal cultures show distinct oscillatory properties

NGN2 forced-overexpression protocols generate a more homogeneous population of predominantly excitatory neurons compared to directed-differentiation protocols, and offer a significantly accelerated differentiation timeline, making them valuable tools for investigating neurological diseases in vitro (Elder et al., 2022; Hulme et al., 2022; Zhang et al., 2013). However, they present several limitations, such as variability in the maturation state of the neurons produced, lack of diverse cortical subtypes, and constraints for modeling neurodevelopmental disorders where it has been proposed that a pathologically primed neural progenitor stage can contribute to disease phenotypes (Hulme et al., 2022; Schafer et al., 2019; Shan et al., 2024).

**Fig. 6.**
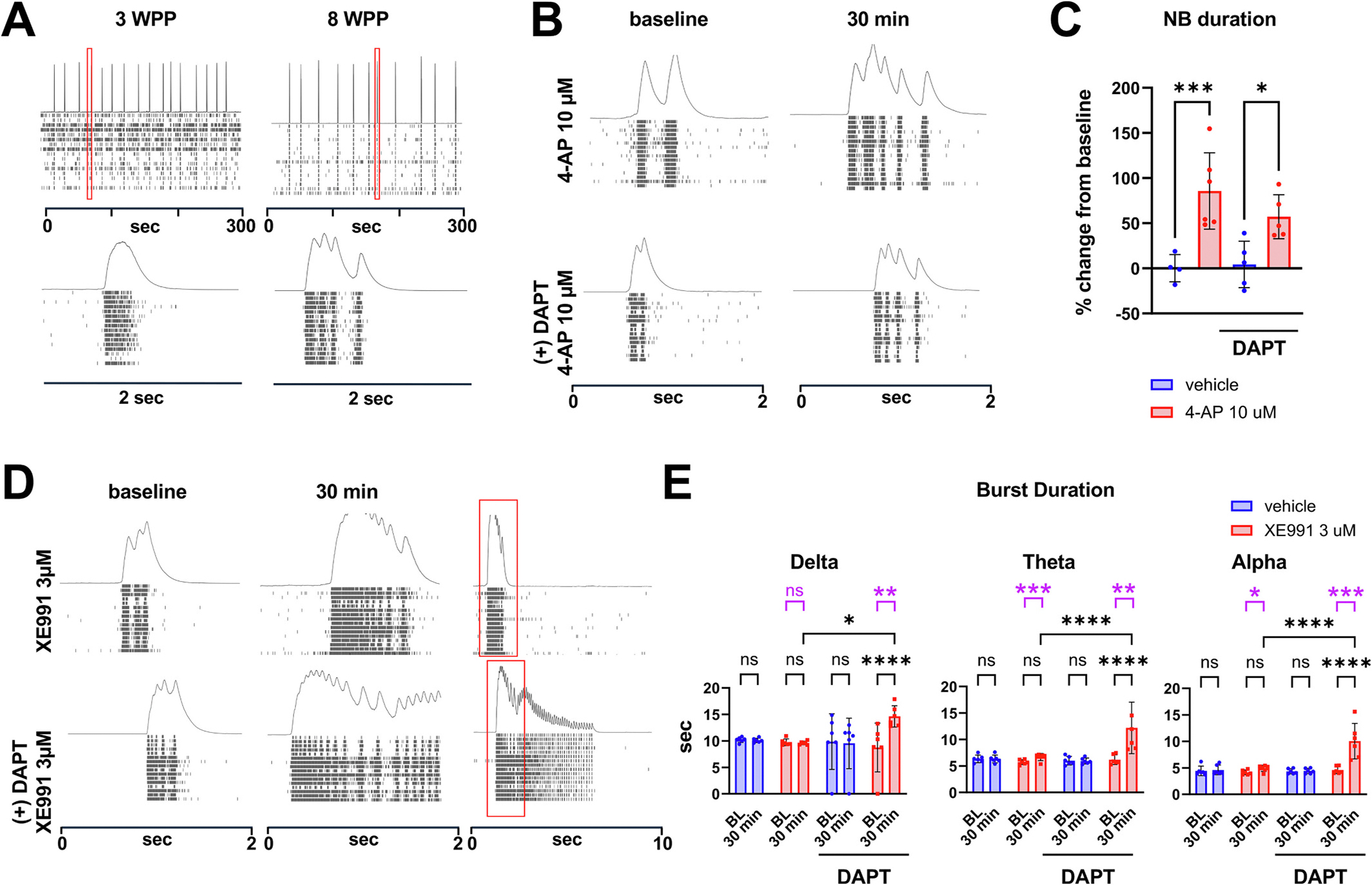
Oscillatory behavior in NGN2-piN in co-culture with PHA. (A) Representative raster plots of MEA recordings from NGN2-piN at 4 and 8 WPP for 300 s (top) and 2 s inset (bottom). (B) Representative raster plots of MEA 2-s recordings from NGN2-piNs treated at 8 WPP with 10 μM 4-AP at baseline and 30 min post-treatment. (C) Network burst duration at 30 min post treatment with vehicle or 4-AP for both plus DAPT and no DAPT conditions for the experiment shown in panel B as % change from baseline. n = 4 wells for no DAPT vehicle, n = 6 wells for no DAPT treated samples, n = 6 wells for both vehicle and treated samples in the plus DAPT condition. Data shown are representative of two independent experiments. *p*-values were calculated using an unpaired t-test. (D) Representative raster plots of MEA recordings from NGN2-piN generated with (+ DAPT) or without the addition of DAPT, shown at baseline (left panels) and at 30 min post treatment with 3 μM XE-991 (middle panels) at 8 WPP. Panels on the right show a 10 s interval at 30 min after treatment, with the red box indicating the 2 s intervals shown in the middle panels. (E) Duration of oscillatory bursts in the delta (1–4 Hz), theta (4–8 Hz), and alpha (8–13 Hz) frequency ranges at baseline and 30 min after treatment with vehicle or XE-991 (3 μM), for both +DAPT and −DAPT conditions corresponding to the experiment in panel D. n = 6 wells. Data are representative of two independent experiments. Two statistical analyses are shown: (1) Black asterisks: a two-way ANOVA with Sidak’s multiple-comparison test comparing all four groups (vehicle and XE-991 in +DAPT and −DAPT conditions) across both timepoints, (2) Purple asterisks: separate two-way ANOVAs with Sidak’s multiple-comparison test performed within each differentiation condition (+DAPT and −DAPT), comparing vehicle vs. XE-991 at baseline and 30 min. All data are shown as mean ± SD and all p-values are indicated as: *0.05 > *p* ≥ 0.01; **0.01 > *p* ≥ 0.001; ***0.001 > *p* ≥ 0.0001; ****p < 0.0001. (For interpretation of the references to colour in this figure legend, the reader is referred to the web version of this article.)

Given these prior studies, we were interested in comparing nested oscillatory behavior in NGN2-induced neurons to our observations from iGluta neurons. To produce NGN2-induced neurons, we turned to a hybrid protocol based on SMAD and WNT inhibition coupled with NGN2 overexpression to produce NGN2-patterned induced neurons (NGN2-piNs) (Nehme et al., 2018), as NGN2-piNs have been previously shown to exhibit nested oscillatory activity (Rasanen et al., 2024). We generated NGN2-piNs from the BioNi010-C-13 hiPSC line (Bioneer A/S), a control line which harbors a doxycycline (Dox) inducible NGN2 cassette in the AAVS1 safe harbor locus (Schmid et al., 2021). We then monitored the development of network organization and the emergence of nested oscillations in NGN2-piNs co-cultured with PHA. We observed that NGN2-piNs exhibited robust, slow (< 1 Hz) network-bursting activity at ~2–3 WPP on the MEA (Fig. 6A), with increased NB frequency compared to iGluta and higher synchronicity, indicated as synchrony index (Fig. S7A). Rudimentary nested oscillations emerged at ~4 to 6 WPP (Fig. 6A), in line with a prior report (Rasanen et al., 2024). Although these rudimentary nested oscillations were detected over extended culture periods as long as 12 WPP, they never developed in terms of duration like those we observed in iGluta, as shown in a comparison with iGluta-alone at 9 WPP and iGluta:iGABA 80:20 at 12 WPP (Fig. S7B and C).

To investigate the viability of NGN2-induced neuronal cultures for testing the contribution of E/I balance to oscillogenesis, we next assessed whether GABAergic neurons affect the rudimentary nested oscillations in these cultures. In contrast to Rasanen et al. (2024), who did not detect GABAergic neurons in their NGN2-piNs cultures, we observed an average of 3.1% ± 1.6 GABA-positive neurons by IF analysis of 3 independent batches (Fig. S7D), likely a reflection of line-to-line differences between our studies. Consistent with the presence of GABAergic neurons in NGN2-piNs cultures, we observed a mild excitatory response to PTX at 8 WPP expressed as number of spikes/NB, NB duration, number of spikes/burst, burst duration, and interspike interval (ISI) coefficient of variation, metrics which collectively reflect network organization (Fig. S7E); however, in contrast to iGluta cultures, the rudimentary nested oscillations we observed in NGN2-piNs cultures were not affected by GABAAR blockade (Fig. S7F), as only one of three experiments showed a small decrease in theta peak power, and there were no detectable changes in delta or alpha peak power or in oscillatory burst duration across all three experiments (Fig. S7G and data not shown). Next, to identify distinct drug-response profiles that may elucidate why NGN2-piNs exhibit less robust nested oscillations than iGluta, thus decreasing their utility for studying oscillogenesis, we investigated the response of NGN2-piNs to 4-AP and XE-991.

NGN2-piNs exhibited an increase in oscillatory burst duration following 4-AP treatment (Fig. 6B, top panel, and Fig. 6C), though the response was less pronounced than that observed in iGluta-alone cultures (Fig. 3), where 4-AP strongly increased oscillatory burst duration and aperiodic-corrected peak power. An intriguing response emerged with XE-991 when NGN2-piNs were differentiated in the presence of DAPT, a Notch inhibitor that promotes cell-cycle exit and synchronized neuronal differentiation. In cultures differentiated without DAPT, XE-991 produced only a modest increase in burst duration within the theta and alpha frequency bands (Fig. 6D and E). In contrast, in +DAPT cultures, XE-991 induced complex nested oscillations and produced a markedly greater increase in burst duration across all frequency bands compared to cultures differentiated without DAPT (Fig. 6D and E). Notably, XE-991 had the opposite effect in iGluta-alone cultures, where it reduced nested oscillatory burst power (Fig. 3), underscoring distinct responses to Kv-channel modulation between the two systems. In contrast, the effect of 4-AP was comparable between plus and minus DAPT conditions (Fig. 6B and C). Together these results suggest that DAPT treatment may influence the responsiveness of Kv7.2/7.3 channels, for instance through altering their expression, heteromeric compositions, cofactors and/or other molecular pathways (Abbott, 2020; Brown and Passmore, 2009; Springer et al., 2021), enhancing the capacity of NGN2-piN cultures to exhibit nested oscillatory behavior.

These findings reveal the capacity of NGN2-piNs cultures to sustain nested oscillations and highlight differences with iGluta cultures in dependence upon GABA and response to Kv channel blockade. Together they underscore the need for further exploration and optimization of NGN2-based differentiation protocols for investigating the physiological basis of oscillogenesis, as transcription factor overexpression strategies offer significant advantages in facilitating efficient and scalable neuron production.

## 3. Discussion

### 3.1. 2D hiPSC neuronal cultures as a viable platform for oscillogenesis studies

The ability to study oscillatory dynamics in vitro using hiPSC-derived neuronal cultures offers significant promise for probing the mechanisms underlying neural network development, function, and dysfunction in a human-relevant context. Oscillatory patterns have been described in most detail in hiPSC-derived 3D brain organoids cultured for prolonged periods of time of >6 months (Samarasinghe et al., 2021; Sharf et al., 2022; Trujillo et al., 2019). Here, we present findings that underscore the potential utility of 2D hiPSC-derived neuronal cultures as simplified systems that are complementary to brain organoids for studying oscillatory activity in vitro, with distinct advantages.

Our results demonstrate that nested oscillations can reliably develop in 2D-cultures of hiPSC-derived glutamatergic neurons derived via directed-differentiation. These nested oscillatory patterns emerged at 3–4 WPP in a culture system consisting of iGluta, a predominantly glutamatergic neuronal population with a subpopulation of GABAergic neurons, co-cultured with hiPSC-derived astrocytes or PHA. We observed oscillations in the delta, theta, and alpha frequency ranges nested within low-frequency (<1 Hz) NBs, which exhibited frequency-specific changes over time. The progression from low-frequency network bursting to more complex, higher frequency nested oscillations highlights a maturation trajectory that mirrors aspects of brain development, in that nested oscillations are reminiscent of “spindle bursts”, characteristic of immature networks in the newborn rodent cortex, or “delta brushes” in the human fetus (Khazipov and Luhmann, 2006; Khazipov et al., 2004; Lamblin et al., 1999; Wu et al., 2024).

We also observed nested oscillatory behavior in 2D cortical cultures generated in-house using a directed-differentiation DSI protocol from a hiPSC line carrying a DISC1 heterozygous mutation (Wen et al., 2014). DISC1 encodes a multifunctional scaffold protein involved in synapse formation and synaptic signaling (Tropea et al., 2018). It has been shown that cortical neurons derived from the patient-specific DISC1 mutant hiPSC that we used in our study exhibit synaptic vesicle-release deficits and dysregulation of synapse-related gene expression, supporting a role in synaptic efficacy (Wen et al., Nature 2014). However, in our study, cortical neurons from this line form active, organized neuronal networks. It is possible that the DISC1 mutation contributed to the oscillatory behavior we observed, but given that nested oscillations also emerged in iGluta cultures on a similar timeline, we think that this is unlikely. However, we note that further studies are needed to identify the conditions and/or factors required to consistently generate cultures that exhibit this behavior, as we have detected robust oscillations in only a limited number of experiments using in-house hiPSC cortical cultures, in contrast to iGluta neurons for which we reliably observe oscillations.

### 3.2. Role of inhibitory neurons and excitatory/inhibitory balance

Interneurons are known to play important roles in generating oscillatory behavior and shaping circuit dynamics (Warm et al., 2021; Wu et al., 2024). Our data reinforce the importance of a functional inhibitory system in modulating network activity and enabling the emergence of nested oscillations. iGluta’s ability to develop nested oscillations, likely mediated by their endogenous GABAergic population, was further modulated by increasing the ratio of GABAergic neurons in iGluta cultures. Thus, the ratio of GABAergic to glutamatergic neurons in the network, or other factors based on whether the glutamatergic and GABAergic neurons are differentiated in the same culture or differentiated separately and then mixed, could be important for tuning oscillatory patterns. Moreover, blocking GABAARs inhibited nested oscillatory behavior across varying glutamatergic/GABAergic neuron ratio conditions, providing additional evidence of inhibitory roles in oscillations in vitro and highlighting the system’s potential for modeling neurological disorders characterized by disrupted E/I balance.

Our results also suggest that SST+ interneurons may play a prominent role in this system, given their prevalence in our cultures and their known involvement in modulating oscillatory behavior during early development. It has been shown that silencing SST+ interneurons during development leads to a reduction in spontaneous and sensory evoked spindle bursts (Baruchin et al., 2022). Replicating this early developmental stage in vitro provides a valuable model for studying the maturation of neural networks and their disruption in neurodevelopmental disorders.

### 3.3. Potassium channel modulation of nested oscillations

Importantly, we demonstrated that nested oscillations in 2D cultures of iGluta neurons can be pharmacologically manipulated through targeted channel blockade to reveal physiological insight into oscillogenesis. For example, we observed broad Kv channel blockade in iGluta cultures and specific blockade of Kv7.2/7.3 channels had opposite effects on nested oscillations, suggesting differential contributions of Kv channel subtypes to oscillatory dynamics, with implications for modeling neurological disorders like epilepsy and neurodevelopmental syndromes, such as KCNQ2/3 encephalopathy (Springer et al., 2021).

Neuronal signals, such as those from the LFP and human electroencephalogram (EEG), comprise mixed periodic and aperiodic components. These components are thought to reflect synchronized and asynchronous neuronal firing in cortical networks, respectively, and have been proposed as putative indices of cortical E/I balance in health and disease (Gao et al., 2017; van Bree et al., 2025). Aperiodic components, characterized by their offset (a measure of broadband power) and exponent (a measure of the slope of the power spectrum), change with healthy aging and show alterations in a wide variety of conditions associated with E/I imbalance, including neurodevelopmental disorders (Ostlund et al., 2022). However, it remains unclear how abnormal cellular network formation gives rise to the reported EEG abnormalities found in neurodevelopmental conditions. In our study, we observed reduced exponents after 4-AP treatment, suggesting a shift in E/I balance toward excitation, presumably due to blocking potassium channels that normally contribute to repolarization and thus limiting excessive firing. These findings build support for using 2D neuronal cultures as model systems to investigate how pharmacological manipulations affect both oscillatory and aperiodic components of neural activity. This approach may provide mechanistic insights into how cellular-level perturbations manifest as the network-level signatures observed in various neurological and psychiatric disorders characterized by E/I imbalance.

### 3.4. Cholinergic modulation of network oscillations

Our findings also reveal a regulatory role for cholinergic modulation of nested oscillations in iGluta neuronal cultures. We extend prior work on the effect of cholinergic modulation of oscillations in rodent adult brain (He et al., 2023; Weiss et al., 2023) and neonatal brain and primary cultures (Hanganu et al., 2007; Kaufman et al., 2012; Kaufman et al., 2014; Kilb and Luhmann, 2003) by revealing more granular effects of cholinergic agonists on oscillatory characteristics. Acetylcholine can alter synaptic transmission via pre- and post-synaptic muscarinic receptors (Fernandez de Sevilla et al., 2021) and can regulate K+ channels, thereby changing neuronal excitability, resulting in depolarization, increased excitability and loss of network organization (Nakajima et al., 1986), which is supported by our observations of disrupted oscillatory activity following carbachol, methacholine, and bethanechol treatment of iGluta neurons. The reorganization we observed after 60 min, suggests involvement of homeostatic mechanisms, a phenomenon also reported in rat primary cortical neuron cultures treated with cholinergic modulators (Kaufman et al., 2012; Kaufman et al., 2014). In carbachol treated cultures, we also found significant reductions of the aperiodic components as reflected by changes in the offset and exponent in the theta and alpha frequency bands and then gradual return toward baseline as the network reorganized, suggesting shifts in a network state that reflects changes in the overall E/I balance.

These parallel changes in both oscillatory and aperiodic components provide a more comprehensive framework for utilizing neural signal characteristics as readouts for plasticity-related processes, offering insights into synaptic remodeling. Moreover, this system holds value for studying diseases characterized by cholinergic deficits that disrupt network dynamics, such as Alzheimer’s disease (Chen et al., 2022b). However, our studies are constrained by limited endogenous acetylcholine in the system.

Interestingly, nested oscillations in iGluta cultures were significantly affected by the mAChR antagonists atropine and scopolamine despite the absence of detectable cholinergic neurons. Potential explanations include constitutive activity of mAChRs (Spalding and Burstein, 2006), as well as the presence of very low levels of choline in the culture medium (Tanaka-Kanegae and Hamada, 2021). It is also possible that they could be due to off-target effects, for instance through inhibition of cAMP-specific phosphodiesterase type 4 (PDE4D) (Perera et al., 2017), or modulation of 5-hydroxytryptamine 3 receptor (5-HT3) (Lochner and Thompson, 2016); however, although PDE4D is expressed in iGluta cultures (see Fig. S2A), rolipram, a PDE4D inhibitor, did not alter nested oscillations in the system (unpublished observation), and we did not detect 5-HT3 expression (see Fig. S2A). Although the mechanism is unclear, these findings are of interest as scopolamine is currently under investigation as a rapid acting antidepressant for the treatment of major depressive and bipolar disorders (Anacker, 2018; Navarria et al., 2015).

### 3.5. Comparison of directed-differentiation with NGN2-induced neuronal models

Our analyses of NGN2-piNs cultures confirmed and extended previous reports of nested oscillatory-like behavior in NGN2-induced cultures (Doorn et al., 2024; Pradeepan et al., 2024; Rasanen et al., 2024; van Hugte et al., 2023) and highlighted differences between this rapid, forced approach and more lengthy directed-differentiation protocols. First, we observed that even though the NGN2-piNs cultures included a small population of GABAergic neurons, GABAAR blockade only impacted the slow (<1 Hz) network bursts without altering the rudimentary nested oscillations we observed. In contrast, studies of 3D neural organoids (Samarasinghe et al., 2021; Sharf et al., 2022; Trujillo et al., 2019) or 2D directed-differentiation neural cultures (Salimpour et al., 2024), including those we describe herein on iGluta, are consistent with roles for a functional inhibitory system in modulating nested oscillations. Thus, further optimization of NGN2-induced neurons and a better understanding of how they compare with those produced via directed differentiation will be necessary if such forced differentiation approaches are to have utility for modeling GABAergic modulation of oscillogenesis that occurs during brain development in vivo.

Second, we observed differential responses of NGN2-piNs and iGluta cultures to the Kv7.2/7.3 channel blocker XE-991. We generated NGN2-piNs under two differentiation conditions, in the absence and presence of DAPT. Treatment of both conditions with 4AP resulted in increased duration of the rudimentary nested oscillatory bursts we observed, albeit not as strongly as we observed for iGluta. However, we observed that treatment of NGN2-piNs cultures with XE-991 caused a mild increase in oscillatory burst duration in the minus DAPT condition but a very strong increase in duration and complexity in the plus DAPT condition, confirming a previous report showing that the Kv7.2/7.3 channel blocker linopirdine increased duration of nested oscillatory-like bursts in DAPT-treated NGN2-iNs cultures (van Hugte et al., 2023). The response of NGN2-piNs to XE-991 is opposite to that seen in iGluta cultures where XE-991 strongly decreased the power of oscillatory bursts. This highlights fundamental differences between the two systems, potentially reflecting variations in Kv7 subtype sensitivity or distinct relationships between potassium channel activity and the regulation of oscillatory dynamics. Interestingly, cortical neurons derived through directed differentiation and NGN2-iNs have been shown to exhibit different developmental expression of KCNQ2 short and long splice variants (Rosa et al., 2020), which are thought to alter Kv7 channel properties (Smith et al., 2001).

Importantly, our observations show that NGN2-piNs have the capacity to sustain complex nested oscillations and suggest that understanding differences in neurons generated with or without DAPT treatment may be key to designing ways to improve the capacity of NGN2-iNs to exhibit nested oscillatory behaviors. For instance, the responsiveness of Kv7.2/7.3 channels could change through altering their expression, heteromeric compositions, and/or co-factors (Abbott, 2020; Brown and Passmore, 2009; Springer et al., 2021). Future optimization of NGN2 protocols would be greatly advantageous to understanding the physiological basis of oscillogenesis given their ease of rapidly producing pure neuronal populations.

### 3.6. 2D versus 3D formats

Three-dimensional (3D) brain organoids provide the most physiologically advanced in vitro representation of human cortical development, achieving higher cellular diversity, laminar organization, and functional maturity than 2D systems, including spontaneous oscillations and synaptic activity reminiscent of fetal or early postnatal brain states (Trujillo et al., 2019; Sharf et al., 2022; Samarasinghe et al., 2021). These features make organoids invaluable for modeling late developmental transitions, gliogenesis, and long-range connectivity. However, their complexity comes at the cost of variability, limited control over cellular composition, and challenges in systematically probing network pharmacology. By contrast, 2D directed-differentiation cultures, although architecturally simple and less mature, enable ease of experimental manipulation, reproducible control of cell-type ratios, and scalable genetic and pharmacological interrogation, including for dose-response analyses, temporal challenges, and mechanistic dissection of ion-channel and receptor function across many replicates. Such scalability allows linking cellular perturbations to quantitative network signatures in ways not currently practicable in organoids. Emerging micropatterned 2D platforms further enhance this potential by imposing spatial organization and localized connectivity while preserving the accessibility required for high-content and electrophysiological assays, offering an alternative to complex organoid assembloids. These complementary approaches, organoids for complexity and maturity, and 2D systems for experimental ease, provide alternative human-relevant models for investigating oscillatory network maturation and its dysregulation in disease, each suited to different experimental aims.

## 4. Conclusions

In vitro models that capture early stages of cortical network maturation, particularly the transition from asynchronous firing to structured, nested oscillatory patterns, are essential for dissecting how developing circuits organize and how this process may be perturbed in disease. Our findings demonstrate that 2D hiPSC-derived neuronal cultures can generate nested oscillations that recapitulate hallmark features of immature cortical activity, including sensitivity to E/I balance. These properties position 2D directed-differentiation systems as experimentally accessible platforms for linking cellular perturbations to emergent network behaviors. At the same time, our results underscore clear developmental constraints of the 2D format. The networks we examined did not progress to later-stage oscillatory regimes such as gamma activity or desynchronization transitions, likely at least in part reflecting the immaturity of the inhibitory compartment and the lack of PV-positive interneurons known to drive these higher-frequency dynamics. Establishing conditions that promote more interneuron maturation, or incorporating defined interneuron subtypes, will be critical for extending this platform to later developmental milestones.

Overall, the capacity of 2D cultures to support nested oscillations, combined with their scalability, pharmacological tractability, and compatibility with controlled cell-type engineering, provides a foundation for future efforts to model oscillatory development and its dysregulation in disease.

## 5. Experimental procedures

### 5.1. Cell culture

All cell culture experiments were conducted in a tissue culture (TC) incubator maintained at 37 °C, 5% CO_2_, and 95% humidity.

### 5.2. Media formulations

#### ES Medium:

DMEM/F12 (Gibco|Thermo-Fisher Scientific) supplemented with 20% (vol/vol) KOSR, 1 mM nonessential amino acids (NEAA) (Invitrogen), 1% Glutamax (Gibco|Thermo-Fisher Scientific), 1% P/S, 0.55 mM 2-mercaptoethanol (Gibco|Thermo-Fisher Scientific) and 10 ng/ml recombinant human FGF2 (R&D Systems).

mTeSR^™^ Plus Medium: mTESR plus basal medium supplemented with 20% mTESR plus 5× supplement (Stemcell Technologies).

#### NBA-K medium:

Neurobasal A medium (Gibco|Thermo-Fisher Scientific) with 10% Knock-out serum replacement (KOSR) (Gibco|Thermo-Fisher Scientific), 1% Penicillin streptomycin (P/S) (Gibco|Thermo-Fisher Scientific), and 1 μg/ml of laminin from Engelbreth-Holm-Swarm murine sarcoma basement membrane (Millipore-Sigma).

BrainPhys Complete medium: BrainPhys (Stemcell Technologies), 2% B27 (Gibco|Thermo-Fisher Scientific), 1% N2 (Gibco|Thermo-Fisher Scientific), 1% P/S.

#### BrainPhys Supplements:

Brain-Derived Neurotrophic Factor (recombinant human/murine/rat BDNF, 20 ng/ml; Peprotech), Glia-Derived Neurotrophic Factor (recombinant human GDNF, 20 ng/ml; Peprotech), ascorbic acid (AA, 200 nM; Sigma Aldrich), dibutyryl cyclic AMP (cAMP, 1 mM, Millipore-Sigma) and laminin (1 μg/ml).

EB medium: DMEM/F12 (Gibco|Thermo-Fisher Scientific) supplemented with 20% KOSR (Gibco|Thermo-Fisher Scientific), 1% NEAA (Invitrogen), 1% Glutamax (Gibco|Thermo-Fisher Scientific), 1% P/S (Gibco|Thermo-Fisher Scientific), supplemented with 2 μM dorsomorphin (Cayman Chemical) and 2 μM A-83 (Tocris Bioscience).

Neural Induction Medium (NIM): DMEM/F12 (Gibco|Thermo-Fisher Scientific): Neurobasal (Gibco|Thermo-Fisher Scientific) (1:1), 1% N2 (Gibco|Thermo-Fisher Scientific), 2% B27 (Gibco|Thermo-Fisher Scientific), 1% NEAA (Invitrogen), 1% Glutamax (Gibco|Thermo-Fisher Scientific), 1% P/S and 2 μM cyclopamine (Cayman Chemical).

#### Neuronal Medium (NM):

Neurobasal medium (Gibco|Thermo-Fisher Scientific) supplemented with 1% Glutamax (Gibco|Thermo-Fisher Scientific), 2% B27 (Gibco|Thermo-Fisher Scientific), 1 μM cAMP (Millipore-Sigma), 200 ng/ml L-Ascorbic Acid (Sigma Aldrich), 10 ng/ml BDNF (Peprotech) and 10 ng/ml GDNF (Peprotech).

#### N2 medium:

DMEM/F12 (Gibco|Thermo-Fisher Scientific) supplemented with 1% N2 (Gibco|Thermo-Fisher Scientific), 0.1% 2-Mercaptoethanol and 1% P/S.

#### Neurobasal-B27:

Neurobasal medium (Gibco|Thermo-Fisher Scientific) supplemented with 2% B27 (Gibco|Thermo-Fisher Scientific), 1% P/S, 10 ng/ml BDNF (Peprotech) and 10 ng/ml GDNF (Peprotech).

### 5.3. Culture of iCell iGluta and iGABA neurons, iCell iAstro astrocytes, and PHA on MEA and imaging plates

To prepare 48-well MEA plates (Axion Biosystems, Atlanta, GA), wells were coated for 1 h with 0.1% poly-ethyleneimine (PEI) (Millipore-Sigma, Burlington, MA) dissolved in borate buffer (Thermo Fisher Scientific). Plates were then washed four times with sterile water and dried overnight. The following day, a 5 μl droplet of 240 μg/ml laminin from Engelbreth-Holm-Swarm murine sarcoma basement membrane (Millipore-Sigma) was applied to each electrode field and incubated for 45 min at 37 °C. We co-cultured iCell GlutaNeurons (iGluta) with either iCell Astrocytes (iAstro) (Fujifilm Cellular Dynamics, Madison, WI) or primary human astrocytes (PHA; ScienCell, Inc.). The choice of astrocyte source depended on practical considerations, including scalability and cost. Comparable neuronal activity was observed in co-cultures with either astrocyte type (see Results). When co-culturing iCell iGluta and iAstro, cells were thawed according to the manufacturer’s protocol and resuspended at an 8:1 ratio in NBA-K medium. A 5 μl droplet containing 100,000 iGluta neurons and 12,500 iAstros was dispensed directly into the laminin-coated electrode field of each well. After 45 min in the incubator, 300 μl of NBA-K medium was added. When iCell GABA neurons (iGABA) were included, they were thawed along with iGluta and iAstro cells and mixed at ratios of either 80:20 (ranging between 75:25 and 85:15) or 50:50 as iGluta:iGABA. The number of iAstro cells remained constant. The resulting cell mixture was dispensed as a 5 μl droplet into each well. Cultures were fed every other day, replacing 66% of the medium with fresh NBA-K medium for the first 5 days. From day 6 onward, the medium was switched to “BrainPhys Complete” plus Brainphys Supplements (Bardy et al., 2015). In the experiments where PHA were used, they were cultured following the supplier’s instructions. When neurons were co-cultured with PHA on MEA plates, they were not added together at the time of plating. Instead, 100,000 PHA cells were added into each well five to seven days after the neurons were plated. Given that the well’s total area is approximately eight times larger than the electrode area, the neuron:astrocyte ratio remained at 8:1. For imaging experiments, iGluta/iAstro and iGABA/iAstro co-cultures were plated at an 8:1 ratio (6000 neurons and 800 astrocytes per well) on 384-well Biocoat poly-D-lysine-coated imaging plates (Corning Life Sciences, Tewksbury, MA) pre-coated with laminin (10 μg/ml) for 2 h. Media transitions for cultures for imaging were conducted as described for MEA cultures.

### 5.4. DSI directed differentiation of iPSCs into cortical neurons and culture on MEA plates

D3–2 iPSCs were differentiated into forebrain neurons as described (Wen et al., 2014). Briefly, iPSCs were cultured on irradiated MEFs in ES medium. Medium was changed daily. For passaging, iPSC colonies were detached from the feeder layer with collagenase IV (Invitrogen) (1 mg/ml) treatment for 1 h, broken-down to small clusters (~100 cells/cluster) and plated on irradiated MEFs. For differentiation, iPSC colonies were detached from the feeder layer with collagenase IV (1 mg/ml) treatment for 1 h and resuspended in EB medium in low attachment plates (Corning) for 4 days with a daily medium change. On day 5, EB medium was replaced by neural induction medium (NIM). After 7 days, the floating EBs were then transferred to reduced growth factor Matrigel (Corning)-coated 6-well plates to form neural tube-like rosettes. The attached rosettes were cultured for 15 days in NIM with medium change every other day. On day 22, the rosettes were picked mechanically and transferred to low attachment plates in NIM. For neuronal differentiation, on day 24 the resuspended neural progenitor spheres were dissociated with Accutase (StemCell Technologies) at 37 °C for 10 min and plated on poly-D-lysine/mouse laminin-coated dishes in NM and half of the medium was changed every other day. After 3 to 4 weeks in culture, differentiated neurons were dissociated using Accutase, filtered through a 70 μm strainer, and plated at a density of 150,000 cells/cm^2^ in NBA-K medium. After 2 days of cytosine arabinoside (5 μM) treatment, neurons were dissociated and plated and cultured on MEA plates (80,000 cells per well) or imaging plates (6000 cells per well) as described in the “Culture of iCell iGluta and iGABA neurons, iCell iAstro astrocytes, and PHA on MEA and imaging plates” section, with addition of PHA at 7 days after plating.

### 5.5. Differentiation of iPSC-derived NGN2-piNs

NGN2-piNs were derived from the BioNi010-C-13 hiPSC line (Bioneer A/S). iPSC were cultured in mTeSR^™^ Plus Medium and passaged every 4 days using Accutase (StemCell Technologies), and plated on reduced growth factor matrigel (Corning) coated plates in mTeSR^™^ Plus Medium with the Rho-associated protein kinase (ROCK) inhibitor Y27632 (Enzo Life Sciences) added at 10 μM, and then maintained in mTeSR^™^ Plus Medium. iPSC were differentiated into neurons using a modified version of a protocol developed by Nehme et al. (2018). Briefly, iPSCs were plated at a density of 100,000 cells/cm^2^ in mTeSR Plus Medium supplemented with 10 μM Y27632 (Enzo Life Sciences) on dishes coated with reduced growth factor matrigel (Corning). The following day, the medium was changed to 10 μM SB431542 (SB) (Tocris), 2 μM XAV939 (XAV) (Stemgent) and 100 nM LDN-193189 (LDN) (Stemgent) in ES medium along with doxycycline hyclate (Dox) (2 μg/ml). Cells were treated with 2 μg/ml Dox until the replating on the final format (MEA or imaging plates). On day 2, the medium was changed to 50% ES medium+SB/XAV/LDN and 50% N2 medium. The following day, the cells were dissociated with Accutase (StemCell Technologies) and plated on Geltrex (Gibco|Thermo-Fisher Scientific)-coated flasks at a density of 80,000/cm^2^ in N2 medium. On day 4, medium was changed to Neurobasal-B27 medium, and then subsequently changed every other day. For some experiments, cells were exposed to 10 μM DAPT (Gibco|Thermo-Fisher Scientific) treatment starting at 4–5 days after Dox induction and until replating. At 7 to 8 days after the addition of Dox, neurons were dissociated with Accutase and replated on MEA plates following the protocol described in the “DSI directed differentiation of iPSCs into cortical neurons and culture on MEA plates” section. Cells were maintained in Neurobasal-B27 medium, with media changes every other day. Five to seven days after plating PHA were added, following the protocol detailed in the “Culture of iCell iGluta and iGABA neurons, iCell iAstro astrocytes, and PHA on MEA and imaging plates” section.

### 5.6. Immunofluorescence and image analysis

Co-cultures plated on 384-well imaging plates were fixed for 15 min at room temperature with 4% paraformaldehyde (PFA; Alfa Aesar Chemicals) in phosphate-buffered saline (PBS; Gibco | Thermo Fisher Scientific). After fixation, cells were washed three times with PBS, permeabilized, and blocked for 30 min using 0.1% Triton-X (Sigma-Aldrich) in blocking buffer (5% donkey serum in PBS; Jackson ImmunoResearch). Primary antibodies directed against MAP2 (Synaptic Systems 188,004), GABA (Sigma A2052), tyrosine hydroxylase (Millipore AB152), and Doublecortin (R&D Systems, AF10025) were diluted in blocking buffer and applied overnight at 4 °C. The following day, cells were washed three times with PBS, and secondary antibodies (Alexa-Fluor; Molecular Probes) were added at a 1:500 dilution in blocking buffer for 2 h at room temperature. After three more PBS washes, cells were incubated with DAPI (0.5 μg/ml; Thermo Fisher) for 30 min at room temperature, followed by additional PBS washes. Images were acquired with the Opera Phenix High Content Screening System (Perkin Elmer, Inc., Waltham, MA) in confocal mode with a 20X water objective. Images of NGN2-piNs were analyzed using Columbus Acapella image analysis software (PerkinElmer). To detect neurons, cells were segmented by first identifying nuclei (“Find Nuclei” building block) and then the cytoplasmic region closely surrounding the nucleus (“Find Surrounding Region”). Intensity of the neuronal marker DCX in the surrounding region was determined (“Calculate Intensity”), and then the neuronal population was detected based on specified threshold intensities (“Select Population”). Sub-populations of DCX positive neurons that co-expressed GABA were identified using the same steps.

### 5.7. Drug treatments

Drugs used in this study were prepared in the following stock concentrations: picrotoxin (PTX, Tocris, 50 mM in DMSO), tiagabine (Selleckchem, 50 mM in DMSO), 4-aminopyridine (4-AP, Sigma-Aldrich, 150 mM in water), XE-991 (Abcam, 100 mM in water), linopirdine (Fisher Scientific, 45 mM in water), carbachol (Sigma, 100 mM in water), acetylcholine (Ach, Fisher Scientific, 100 mM in water), nicotine (Sigma, 30 mg/ml in DMSO), methacholine (Combi-Blocks, 100 mM in water), atropine (Sigma, 30 mM in water), scopolamine (Tocris, 30 mM in water), rolipram (ROL) (Tocris, 50 mM in DMSO). Drugs were used at the following final concentrations: PTX at 50 μM; tiagabine at 3 μM, 4AP at 10 μM, XE-991 at 3 μM, linopirdine at 0.5 μM, carbachol at 3 μM, ACh at 1 mM, nicotine at 30 μM, methacholine at 1 μM, atropine at 0.5 μM, scopolamine at 0.5 μM, ROL at 50 μM. Twenty-four hours before treatment, 66% of the medium was replaced, and this step was repeated two hours before treatment. Baseline activity was recorded prior to drug addition. Wells were assigned to treatment or control groups based on their baseline activity (e.g., WMFR and number of NBs) to ensure comparable activity levels across groups. Drugs were diluted to a 30× concentration in 10μl of medium and added directly to wells. Recordings were performed 5, 30, and 60 min after treatment unless otherwise specified. Following the final recording, drugs were washed out using serial medium exchanges. Twenty-four hours post-washout, plates were re-recorded to assess recovery.

### 5.8. Multi-electrode array recording and data analysis

Cell plating densities were consistent across experiments (100,000 for iGluta, and 80,000 for NGN2-piNs and directed-differentiated cortical neurons). Activity was consistent across the plates, as indicated by the average number of active electrodes at 3–4 WPP (11.39 ± 2.56, *n* = 15 plates for iGluta; 15.69 ± 0.48, *n* = 4 plates for NGN2-piNs; 10.35 ± 0.5 for in house DSI cortical neurons, *n* = 2 plates).

Recordings were performed using the Maestro system with Axion Integrated Studio software (Axion Biosystems). Data acquisition was conducted at 37 °C with a Butterworth band-pass filter (10–2500 Hz) and adaptive spike threshold set to 5.5× standard deviations.

Two parameter sets were applied for analysis: (1) Burst and Network Burst Parameters: Active electrode criterion: ≥5 spikes/min; Burst detection (Poisson surprise): Min surprise = 5; Network bursts: Envelope threshold factor = 2, Min IBI = 1000 ms, Min electrodes = 25%, Burst inclusion = 75%; Synchrony window = 20 ms. (2) Nested oscillations: Active electrode criterion: ≥5 spikes/min; Burst detection (ISI threshold): Min spikes = 5, Max ISI = 100 ms; Network bursts: ISI threshold with min spikes = 10, Max ISI = 10 ms, Min electrodes = 25%; Synchrony window =20 ms. Data were analyzed using Excel (Microsoft) and GraphPad Prism version 10 (GraphPad Software).

In this study, we defined specific patterns of coordinated neuronal activity based on their temporal characteristics. We use the term “network bursts” to refer to slow, coordinated population activity occurring at frequencies below 1 Hz (typically 0.3–0.4 Hz in our recordings). Within these network bursts, we observed faster rhythmic activity in the 1–13 Hz range, which we term “nested oscillations”, which encompassed the delta (1–4 Hz), theta (4–8 Hz), and alpha (8–13 Hz) frequency bands.

### 5.9. Signal processing and analysis

All signal processing and analyses were performed using custom Python (v3.9) scripts and ran on a Windows 10 workstation. Raw spike times were sampled from all 16 channels per well at 12.5 kHz and were downsampled by binning spikes into 2 ms windows, resulting in a final histogram sampling rate of 500 Hz. To generate a continuous signal from discrete spike events, we employed a kernel-based approach (Telenczuk et al., 2020) and convolved binned spike times from all electrodes with a double-exponential kernel parameterized to mimic glutamatergic postsynaptic responses (τ_rise_ = 2 ms, τ_decay_ = 25 ms, kernel_window = 300 ms). This approach transformed discrete spiking events into a continuous waveform that captures the temporal dynamics of population activity, similar to methods used to approximate LFPs in computational models where each spike contributes to the extracellular field through its postsynaptic effects. This kernel-smoothed signal served as our basis for subsequent frequency-specific analyses and burst detection (Ness et al., 2025). Band-specific oscillation detection was performed to quantify temporal patterns in oscillatory activity across three frequency bands: delta (1–4 Hz), theta (4–8 Hz), and alpha (8–13 Hz). For each band, the histogram was bandpass filtered using a FIR filter and the instantaneous amplitude envelope was calculated using the Hilbert transform. The amplitude envelope was smoothed using a Gaussian filter with a sigma parameter that scaled with both the center frequency and bandwidth of each frequency band. Oscillation detection used a dual threshold crossing method with thresholds set at the 50th and 75th percentiles of the smoothed amplitude envelope. To be classified as a nested oscillation, the signal had to exceed both the low and high thresholds, with the high threshold being crossed at least once during the candidate oscillation duration. Each frequency band had specific minimum cycle requirements (delta: 8 cycles, theta: 16 cycles, alpha: 24 cycles) and allowances for brief amplitude drops (delta: 3 cycles, theta: 4 cycles, alpha: 5 cycles) for candidate oscillations. For oscillations lasting at least one second, spectral analysis was performed using Welch’s method with 4-s segments and 2-s overlap, analyzing frequencies from 0.05 to 40 Hz. Individual spectra were parameterized using *specparam* (v 2.0.0) (peak width limits 1–8 Hz, minimum peak height 0.2, maximum 4 peaks, peak threshold 1.5, fixed aperiodic mode). Within each frequency band, peak frequency and power were measured at the maximum point of the aperiodic-adjusted (“flattened”) spectrum. For each frequency band, we quantified mean nested oscillation duration, number of nested oscillation events, nested oscillation occurrence rate, and spectral characteristics including mean peak frequency, mean aperiodic-corrected peak power, mean offset, and mean exponent.

### 5.10. RNA-seq analysis

Triplicate samples of iGluta neurons were prepared 3 weeks after plating. Total RNA was isolated using a RNeasy mini kit (Qiagen, 74104). Invitrogen Qubit and Agilent TapeStation were used to determine RNA concentration and RNA integrity numbers respectively prior to library preparation. PolyA RNA was isolated using the NEBNext^®^ Poly(A) mRNA Magnetic Isolation Module and barcoded libraries were made using the NEBNext^®^ Ultra II^™^ Directional RNA Library Prep Kit for Illumina^®^ (NEB, Ipswich MA). Barcoded libraries were pooled and sequenced as paired-end 151 bp reads on an Illumina NovaSeq platform. Bulk RNA-seq samples were processed using nf-core rnaseq pipeline version 3.17.0 using parameters “–aligner star_rsem” to perform read trimming, quality control, alignment, gene quantification, and normalization. We used human genome version GRCh38.p14 sequence and GENCODE gene annotations version 47.

### 5.11. Statistical analyses

All statistical tests and sample sizes are included in the figure legends and text. All data are shown as mean ± SD as stated in the figure legends. In all cases, the *p* values are represented as follows: *0.05 > *p* ≥ 0.01; **0.01 > *p* ≥ 0.001; ***0.001 > *p* ≥ 0.0001; ****p < 0.0001 and ns, not statistically significant when *p* > 0.05. Repetitions of independent experiments are on different batches, and each independent experiment has a stated number of wells per condition. With the exception of Fig. 2A, in all cases, the stated “n” value is the number of separate wells, whereas in Fig. 2A the stated n are plates, for which the numbers of wells are also stated. With the exception of Fig. 2A, statistical analyses were performed using the GraphPad Prism 9 software. See Table S1 for F values and degrees of freedom used for ANOVA calculations. Cases where a mixed-model was used are noted in this table. For Fig. 2A, statistical analysis was performed using Python 3.10 (statsmodels) implementing a generalized estimating equation (GEE) model. The dependent variable was the fraction of wells exhibiting oscillatory activity per plate and timepoint. Because this outcome represents a bounded proportion (0–1) derived from binary well-level events (with vs. without nested oscillations), the data follow a binomial rather than normal distribution and include repeated measures across time for each plate. Consequently, wells with nested oscillation fractions were analyzed using a generalized estimating equation (GEE) with a binomial logit link and an exchangeable within-plate correlation structure to account for non-independence of repeated observations. The model included fixed effects for neuronal composition (iGluta-alone, 80:20 iGluta:iGABA, or 50:50 iGluta:iGABA), time (weeks, centered), and their interaction. Because plates contributed variable numbers of wells, the total number of wells per plate was incorporated as an observation-level weight, ensuring that plates with more wells, which provide more precise estimates of oscillation probability, contributed proportionally more to the fit. The fitted model can be expressed as: “logit”(p_ij) = β_0 + β_1 t_ij + β_2 C_80 + β_3 C_50 + β_4 (t_ij × C_80) + β_5 (t_ij × C_50), where p_ij is the probability that a well oscillates in plate i at week j, t_ij is the centered time variable (week), and C_80 and C_50 are indicator variables for the 80:20 iGluta:iGABA, or 50:50 iGluta:iGABA conditions (iGluta-alone = reference). Model coefficients (β _±_ SE) were evaluated using Wald χ^2^ statistics, and results are reported as odds ratios (OR [95% CI]) derived from exponentiated coefficients. See Table S1 for full model parameters and odds ratios.

## Supplementary Material

1

2

## Data Availability

Links to data and code are provided in the manuscript

## Appendix A. Supplementary data

Supplementary data to this article can be found online at https://doi.org/10.1016/j.nbd.2026.107281.
