# Supplementary material for: Pharmacological manipulation of nested oscillations in human iPSC-derived 2D neuronal networks
Source: Neurobiol Dis. Author manuscript; Available in PMC 2026 Apr 27. (PMC13110928; doi:10.1016/j.nbd.2026.107281)
Supplement: 1 [file NIHMS2151752-supplement-1.pdf]

Supplementary Information

SUPPLEMENTARY FIGURE LEGENDS

Figure S1. Characterization of primary human astrocytes (PHA) and iAstro and comparison between astrocyte addition protocols on MEA

(A-D) Representative confocal images of primary human astrocytes **(A)** and iAstro **(B)**, immunostained for the astrocyte markers GFAP (green) (channels uncoupled in A′ for PHA and B′ for iAstro) and CD44 (red) (channels uncoupled in A″ for PHA and B″ for iAstro). Negative staining for the neuronal marker Tau (gray) confirms the absence of neuronal cells (shown separately in A‴ for PHA and B‴ for iAstro). Nuclei are detected with DAPI (blue). **(C)** iGluta-alone stained with Tau as a control in the same experiment shown in (A) and (B). **(D and E)** For reference, iGluta/astrocyte co-cultures immunostained for the neuronal marker MAP2 (green) and astrocyte marker GFAP (red), with nuclei detected with DAPI (blue): iGluta+PHA **(D)** and iGluta+iAstro **(E)**. Scale bar is 50 µm for all images. **(F)** Representative raster plots of MEA recordings at 3 WPP from iGluta neurons co-cultured with astrocytes using two different protocols: (left) co-plating with iAstro at day 0 (iAstro D0), or (right) addition of primary human astrocytes (PHA) at day 7 after neuronal plating (PHA D7). Both conditions show comparable levels of activity and network organization. **(G)** Quantification of weighted mean firing rate (WMFR) (left panel), calculated by weighting the mean firing rate of each individual electrode by the total number of active electrodes in that well, number of NB (right panel) of the experiments shown in F, showing no significant differences in iGluta co-cultures generated with the two astrocyte addition protocols. n= 15 wells for iAstro D0, n=48 wells for PHA D7. Data are shown as mean ± SD. *p*-values were calculated using an unpaired t-test and are indicated as: $*0.05 > p \geq 0.01$; $**0.01 > p \geq 0.001$; $***0.001 > p \geq 0.0001$; $****p < 0.0001$.

Figure S2. Characterization of the cellular composition of iGluta and iGABA cultures

(A) Heatmap of gene expression indicated as Log2 (TPM+1) transformed values of three replicates of iGluta neurons at 3 WPP, confirming that the iGluta cell population is a mixture of primarily glutamatergic neurons, including lower layer TBR1 expressing cortical neurons, and GABAergic neurons. ChAT expression is barely detectable, consistent with a lack of detectable ChAT positive neurons in the culture in IF analyses (data not shown). **(B-D)** Representative confocal images of iGluta/iAstro (B and C) and iGABA/iAstro (D) co-cultures immunostained at 2 WPP with the dendritic marker MAP2 (green) and the GABAergic neuron marker GABA (red) (B and D), and at 4 WPP with the cortical lower layer neuronal marker TBR1 (red) (C). Nuclei are detected with DAPI (blue) (B-D). Scale bar is 50 µm for all images. Panels B' and D' show channels uncoupled from merges in panels B and D.

Figure S3. Description of algorithm for detecting and quantifying nested oscillations

Analysis of multi-unit burst activity and frequency-specific oscillatory bursts from MEA recordings. **(A)** Raster plot showing simultaneous spiking activity across 16 channels over recording period. **(B)** Population activity histogram generated by convolving spike times with a double exponential kernel to model glutamatergic postsynaptic responses. **(C-E)** Frequency band analysis is shown for delta (1-4 Hz), theta (4-8 Hz), and alpha (8-13 Hz), respectively, for example bursts. Each band shows the band-filtered signal at the top, followed by amplitude envelopes where detected bursts are shown in red, with high (green) and low (cyan) detection thresholds. Below these, power spectral analyses show the original spectrum (black), full *specparam* model fit (red), and estimated aperiodic component (blue). The bottom panels show aperiodic adjusted power spectra with aperiodic-corrected peak power and frequencies marked by red asterisks. Gray shaded regions in bottom panels indicate the frequency band of interest.

Figure S4: Oscillatory behavior in hiPSC-derived cortical neurons differentiated through dual SMAD inhibition

(A-C) Representative confocal images of hiPSC-derived cortical neurons (differentiated in-house using a DSI protocol) in co-culture with PHA immunostained for MAP2 and TBR1

(A), GABA (B) and GFAP (C). **(D)** Nested oscillations are observed at 4 WPP as shown in the representative rasterplot of a 300 second recording with the red box indicating a 5-second interval inset shown in the right panel. **(E-F)** The addition of PHA significantly modulates nested oscillations, as evidenced by an increased delta frequency peak (E) and enhanced mean power in the theta and alpha frequency bands at 4 WPP (F). n = 23 wells for (+) PHA and n = 24 wells for (-) PHA. **(G)** Quantification of oscillatory burst parameters (peak frequency, peak power, burst duration and oscillatory burst percentage) in the delta, theta and alpha frequency ranges for an independent batch of hiPSC-derived cortical neurons differentiated in-house using a DSI protocol in co-culture with PHA. n=6 wells. All data are shown as mean ± SD. *p*-values were calculated using an unpaired t-test and are indicated as: $*0.05 > p \geq 0.01$; $**0.01 > p \geq 0.001$; $***0.001 > p \geq 0.0001$; $****p < 0.0001$.

Figure S5.

Analyses of inhibitory input in iGluta and iGluta/iGABA co-cultures

(A) Representative raster plots of MEA recordings from iGluta/iAstro treated with vehicle or 50 μM PTX at baseline and 5 minutes post-treatment at 5 WPP. **(B and C)** Quantification of responses of iGluta/iAstro and iGluta:iGABA/iAstro cultures with 80:20 and 50:50 iGluta:iGABA to drug treatments expressed as % change from baseline. **(B)** Quantification of key parameters from PTX treatment in panel A, percent spikes in bursts (burst %), median inter-spike interval (ISI) within network bursts, and synchrony index. n = 6 wells for 80:20 PTX-treated samples and 8 wells for all other samples per condition. **(C)** Quantification of responses to 3 μM tiagabine at 5 minutes post-treatment, showing synchrony index and percent spikes in network burst (NB%) at 4 WPP. n = 5 wells for iGluta-alone samples, and 6 wells for all other samples, per condition. The data are representative of 5 independent experiments for A-B and 2 independent experiments for C. **(D)** Representative raster plots showing the effect of 50 μM PTX on nested oscillations in iGluta/iAstro co-cultures and iGluta:iGABA/iAstro co-cultures at a ratio of 80:20 and 50:50 iGluta:iGABA at 6 WPP. All data are shown as mean ± SD. *p*-values were calculated using two-way ANOVA with Tukey's multiple comparisons test and are indicated as $*0.05 > p \geq 0.01$; $**0.01 > p \geq 0.001$; $***0.001 > p \geq 0.0001$; $****p < 0.0001$.

Figure S6. Additional experiments with Kv7 channel and mAChR modulators

(A) Representative raster plots of iGluta/PHA co-cultures at baseline and 30 minutes post-treatment with XE-991 (3 μM) at 6 WPP. **(B)** NB frequency and peak power (alpha and theta frequency range) at baseline and 30 minutes for XE-991-treated iGluta cultures at 6 WPP, for the experiment shown in panel A. n=5 wells for vehicle, n=6 wells for XE-991-treated samples. **(C)** Representative raster plots of iGluta/PHA co-cultures at baseline, 5-and 60-minutes post-treatment with Bethanechol (100 μM) at 3 WPP. **(D-E)** Quantification of NB% (D) and NB duration (E) calculated as % change from baseline at 5 and 60 minutes after treatment with bethanechol for the experiment shown in panel C. n=6 wells for vehicle and drug treated samples. All data are shown as mean ± SD. *p*-values were calculated using two-way ANOVA with Sidak's multiple comparison test (B and D), and an unpaired t-test (E), and are indicated as: $*0.05 > p \geq 0.01$; $**0.01 > p \geq 0.001$; $***0.001 > p \geq 0.0001$; $****p < 0.0001$.

Figure S7. **Analysis of NGN2-piNs.**

(A) Comparison of NB frequency and synchrony index between NGN2-piN/PHA and iGluta/iAstro cultures at 6 WPP. n = 11 wells for NGN2-piNs and n=13 wells for iGluta. **(B)** Representative raster plots of NGN2-piN and iGluta at 9 WPP showing a 120-second recording and a 5-second inset, and quantification of NB duration for the experiment shown in the same panel. n=12 wells for NGN2-piNs and n=5 wells for iGluta. **(C)** Representative raster plots of NGN2-piN and 80:20 iGluta:iGABA at 12 WPP showing a 120-second recording and a 5-second inset, and quantification of NB duration for the experiment shown in the same panel. n=7 wells for NGN2-piNs and n=8 wells for iGluta:iGABA co-culture. **(D)** Characterization of GABAergic neurons in NGN2-piN. Neurons were immunostained at 2 WPP with the neuronal marker Doublecortin (DCX, green), the GABAergic neuron marker GABA (red), and the DNA stain DAPI to label the nuclei (blue). Scale bar is 50 μm. An average of 3.1% ± 1.6 of GABAergic neurons was detected in the cultures, calculated over 3 independent batches and a total of 325 fields and 37512 neuronal nuclei. **(E)** Quantification of response of NGN2-piN/PHA co-cultures 5 minutes post-treatment with 50 μM PTX for number of spikes/NB, NB duration, number

of spikes per burst, burst duration, and interspike interval (ISI) coefficient of variation expressed as % change from baseline at 8 WPP. Metrics collectively indicate increased network organization in response to PTX. n = 12 wells for vehicle and n=10 wells for PTX treated. Data shown are representative of three independent experiments. **(F)** Representative raster plots of 2-second recordings from NGN2-piN at baseline and 5 minutes post 50 μM PTX treatment. **(G)** Analysis of oscillatory burst peak power in the theta frequency range in NGN2-piN cultures at 8 WPP, 5 minutes after treatment with PTX (50 μM) for three independent experiments. Batch 1: n=6 wells vehicle and PTX-treated; batch 2: n = 12 wells for vehicle and n=10 wells for PTX-treated; batch 3: n=8 wells vehicle and n=4 wells PTX-treated. All data are shown as mean ± SD. *p*-values were calculated using an unpaired t-test (A, B, C and E) and two-way ANOVA with Sidak's multiple comparison test (G) and are indicated as: ∗0.05 > p ≥ 0.01; ∗∗0.01 > p ≥ 0.001; ∗∗∗0.001 > p ≥ 0.0001; ∗∗∗∗p < 0.0001.

Table S1 (Excel file) F values and degrees of freedom used for ANOVA calculations and model parameters and odds ratios for GEE. Table S1_ F values and GEE model parameters.xlsx

Fig. S1

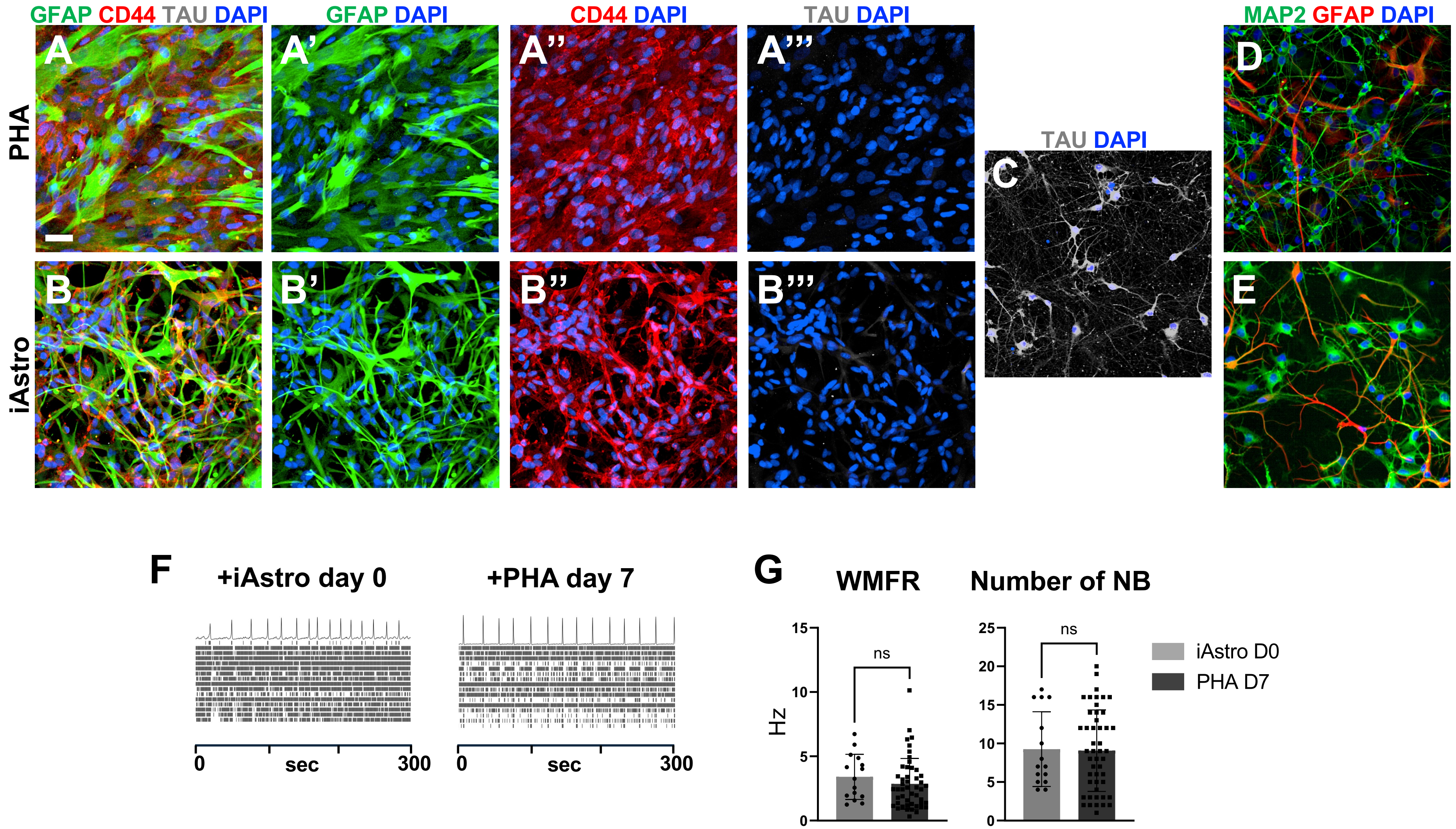

GFAP CD44 TAU DAPI
GFAP DAPI
CD44 DAPI
TAU DAPI
TAU DAPI
MAP2 GFAP DAPI
A
A'
A''
A'''
B
B'
B''
B'''
C
D
E
PHA
iAstro
F
+iAstro day 0
+PHA day 7
0
sec
300
G
WMFR
Number of NB
Hz
ns
iAstro D0
PHA D7

Fig. S2

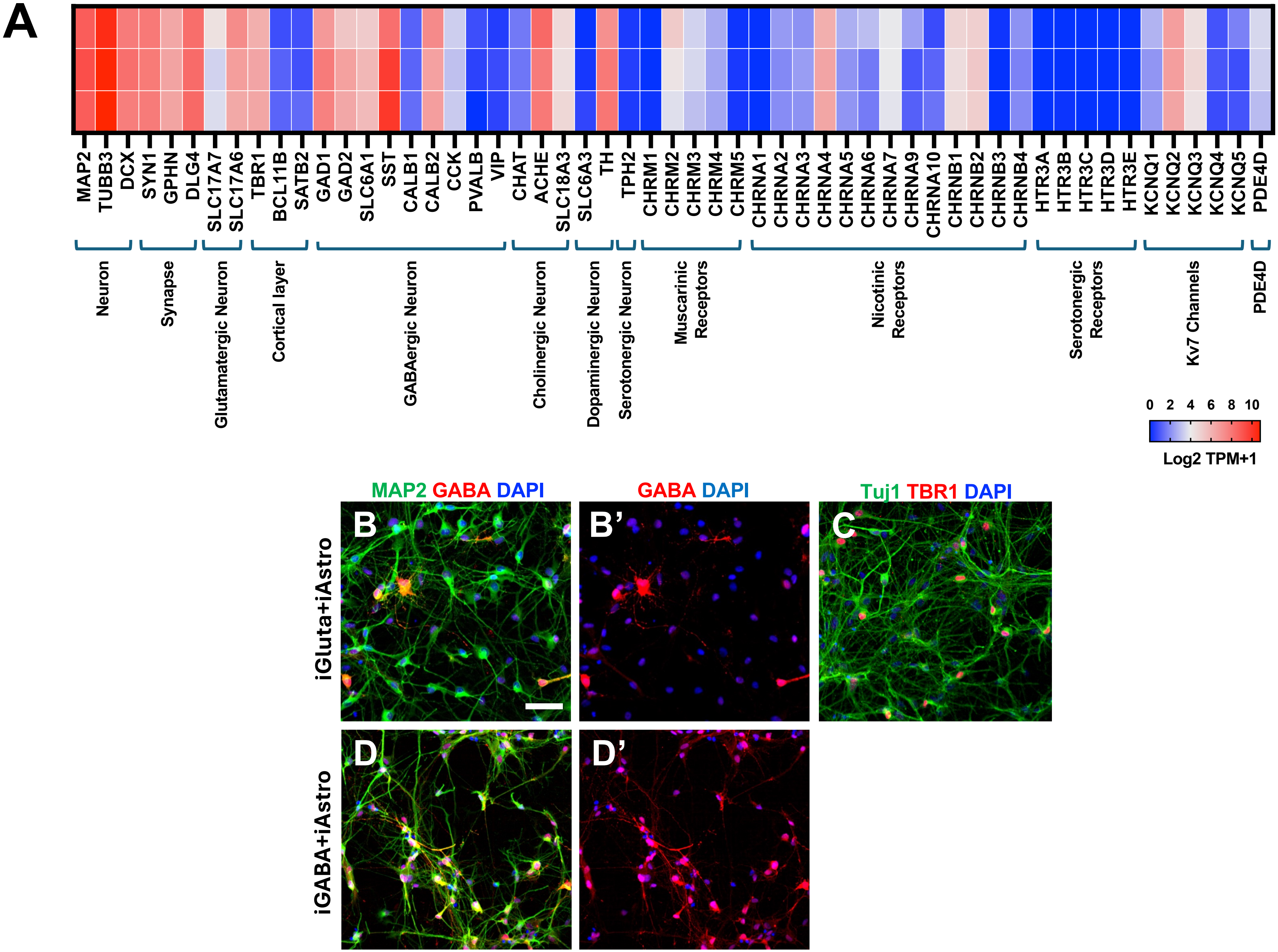
A
MAP2
TUBB3
DCX
SYN1
GPHN
DLG4
SLC17A7
SLC17A6
TBR1
BCL11B
SATB2
GAD1
GAD2
SLC6A1
SST
CALB1
CALB2
CCK
PVALB
VIP
CHAT
ACHE
SLC18A3
SLC6A3
TH
TPH2
CHRM1
CHRM2
CHRM3
CHRM4
CHRM5
CHRNA1
CHRNA2
CHRNA3
CHRNA4
CHRNA5
CHRNA6
CHRNA7
CHRNA9
CHRNA10
CHRNB1
CHRNB2
CHRNB3
CHRNB4
HTR3A
HTR3B
HTR3C
HTR3D
HTR3E
KCNQ1
KCNQ2
KCNQ3
KCNQ4
KCNQ5
PDE4D
Neuron
Synapse
Glutamatergic Neuron
Cortical layer
GABAergic Neuron
Cholinergic Neuron
Dopaminergic Neuron
Serotonergic Neuron
Muscarinic Receptors
Nicotinic Receptors
Serotonergic Receptors
Kv7 Channels
PDE4D
0 2 4 6 8 10
Log2 TPM+1
MAP2 GABA DAPI
GABA DAPI
Tuj1 TBR1 DAPI
B
B'
C
iGluta+iAstro
D
D'
iGABA+iAstro

Fig. S3

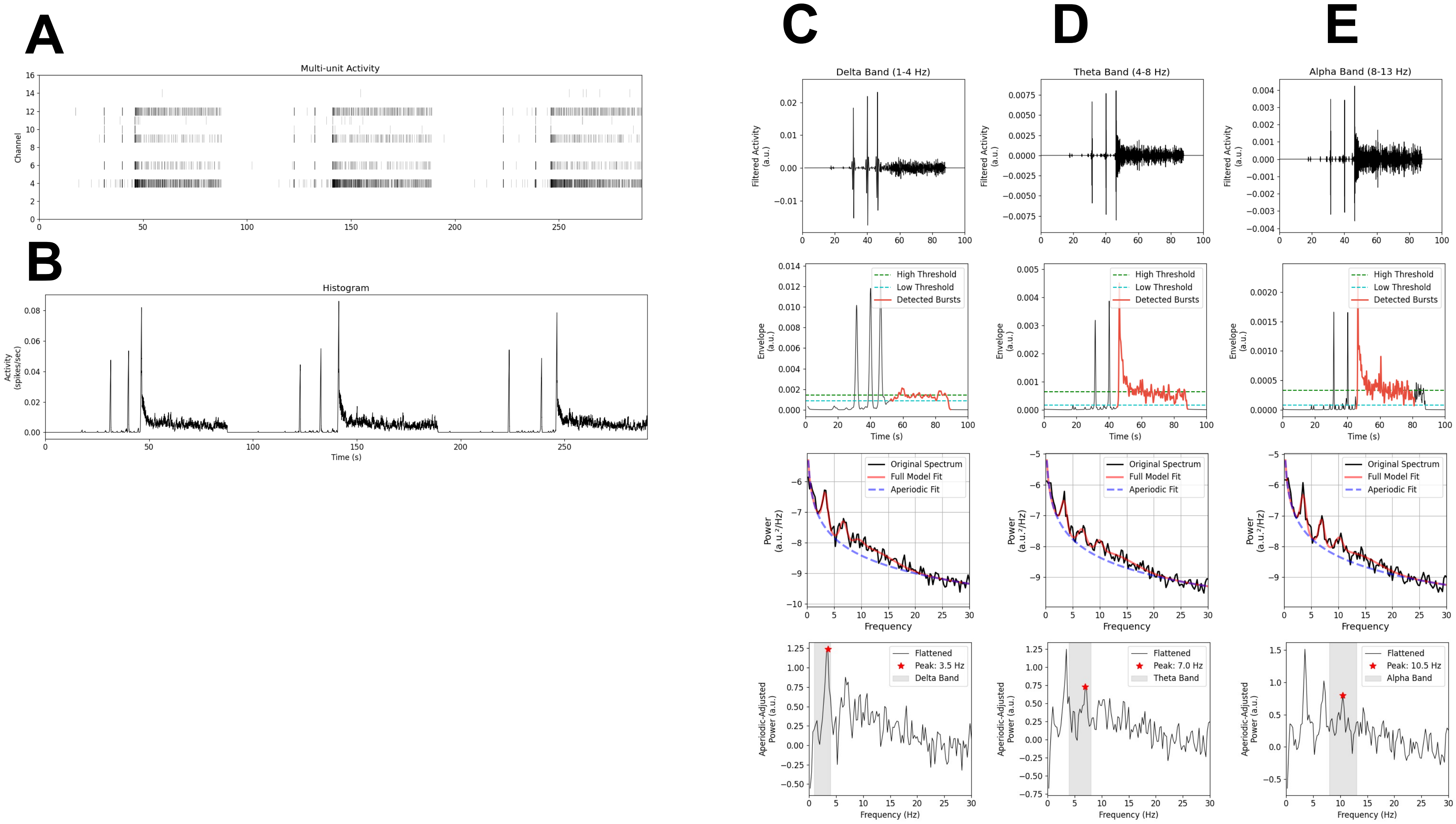
A
Multi-unit Activity
Channel
B
Histogram
Activity (spikes/sec)
Time (s)
C
D
E
Delta Band (1-4 Hz)
Theta Band (4-8 Hz)
Alpha Band (8-13 Hz)
Filtered Activity (a.u.)
Envelope (a.u.)
High Threshold
Low Threshold
Detected Bursts
Time (s)
Power (a.u.²/Hz)
Original Spectrum
Full Model Fit
Aperiodic Fit
Frequency
Aperiodic-Adjusted Power (a.u.)
Flattened
Peak: 3.5 Hz
Delta Band
Peak: 7.0 Hz
Theta Band
Peak: 10.5 Hz
Alpha Band
Frequency (Hz)

Fig. S4

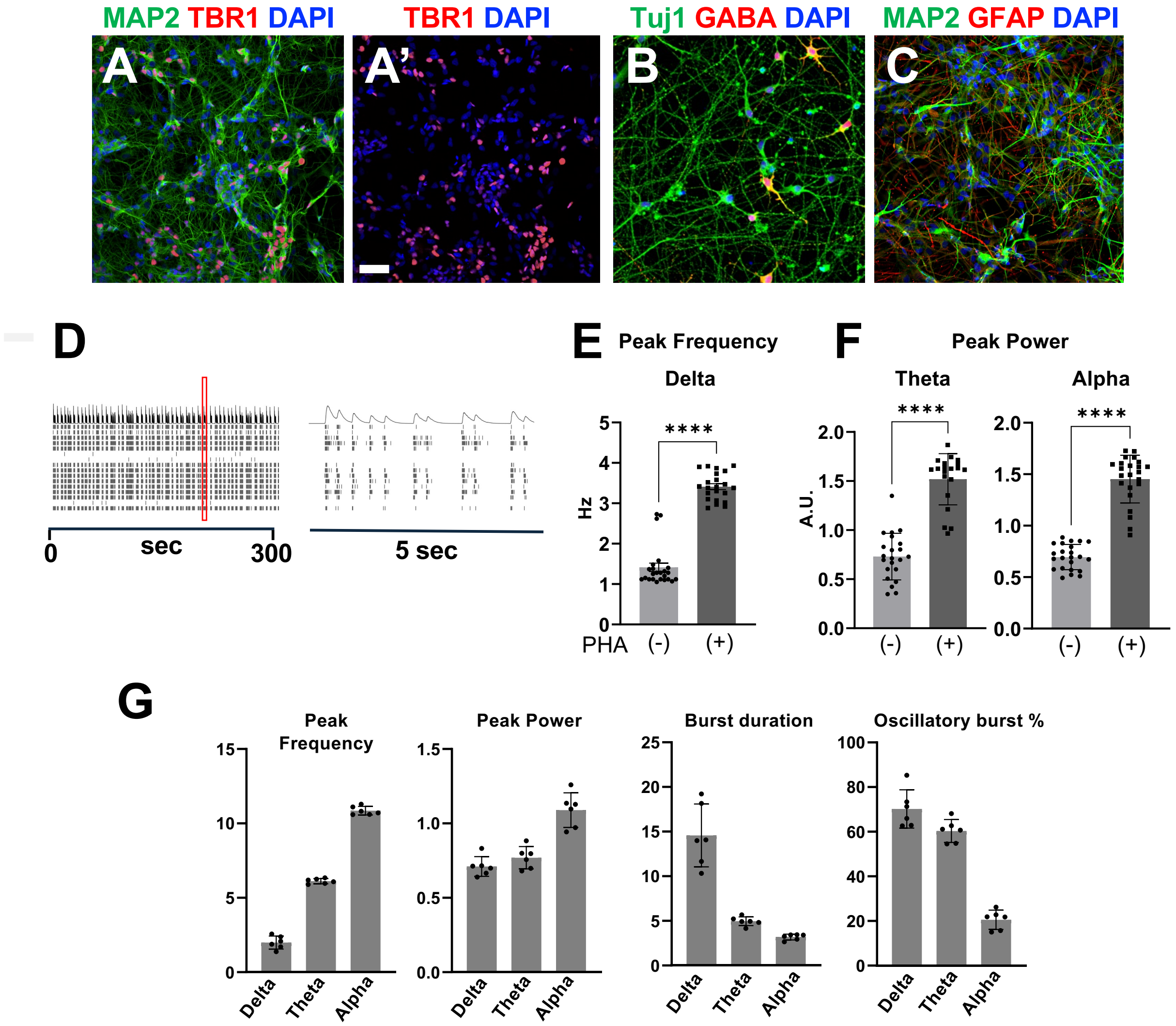

MAP2 TBR1 DAPI
TBR1 DAPI
Tuj1 GABA DAPI
MAP2 GFAP DAPI
A
A'
B
C
D
0
sec
300
5 sec
E
Peak Frequency
Delta
Hz
****
PHA
(-)
(+)
F
Peak Power
Theta
Alpha
A.U.
****
G
Peak Frequency
Peak Power
Burst duration
Oscillatory burst %
Delta
Theta
Alpha

Fig. S5

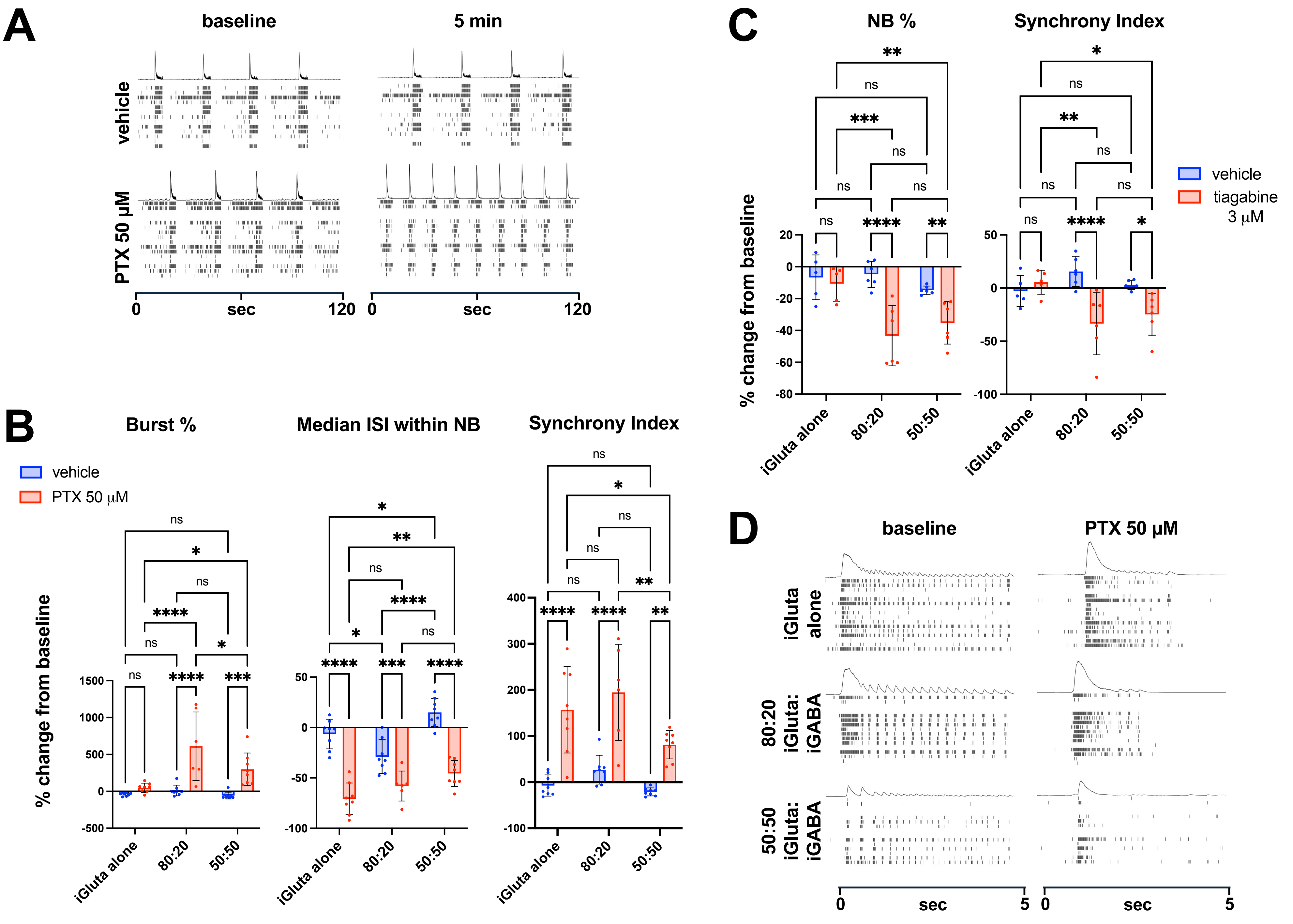

A
baseline
5 min
vehicle
PTX 50 µM
0
sec
120
B
Burst %
Median ISI within NB
Synchrony Index
vehicle
PTX 50 µM
% change from baseline
iGluta alone
80:20
50:50
C
NB %
Synchrony Index
vehicle
tiagabine 3 µM
% change from baseline
iGluta alone
80:20
50:50
D
baseline
PTX 50 µM
iGluta alone
80:20 iGluta: iGABA
50:50 iGluta: iGABA
0
sec
5

Fig. S6

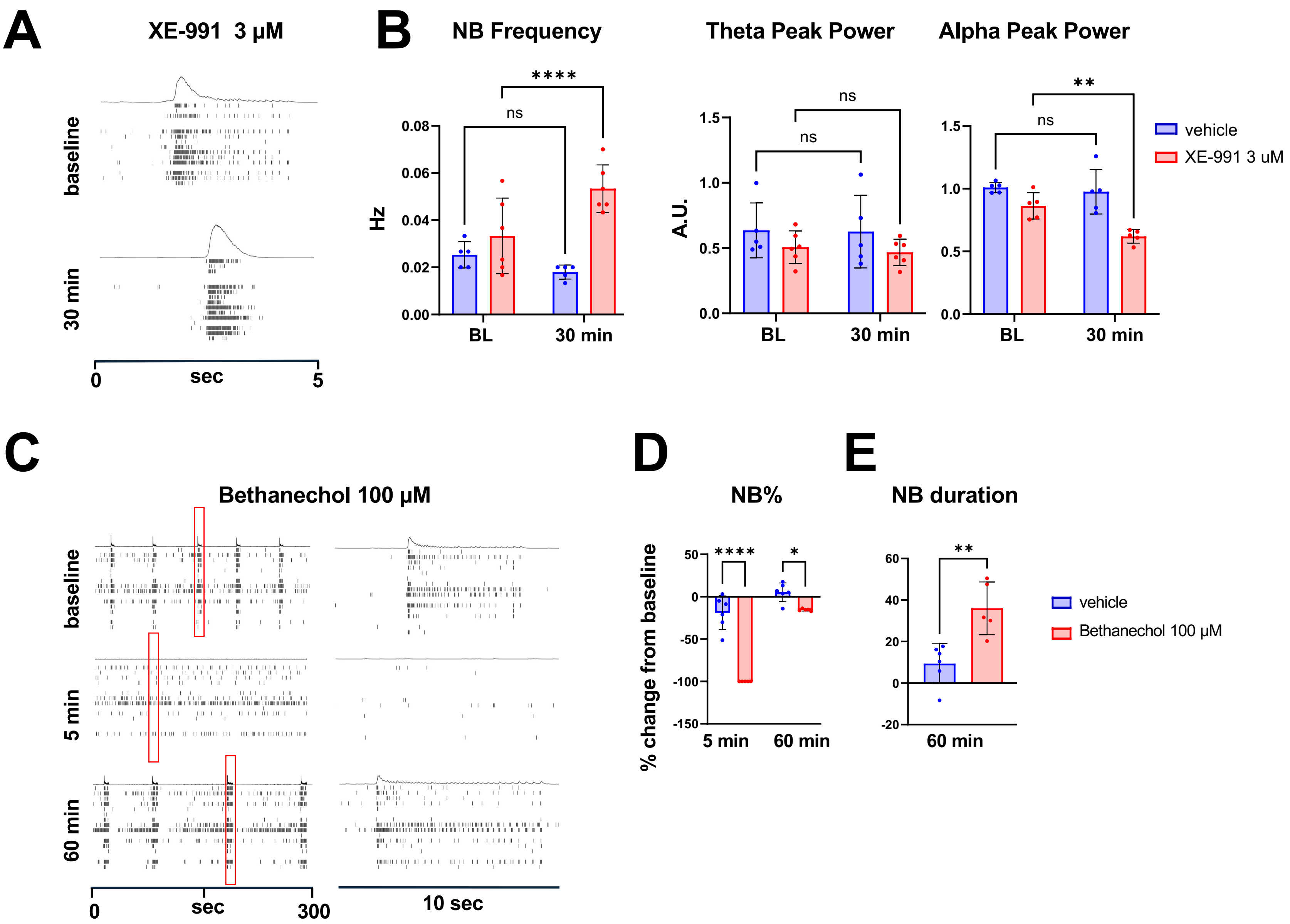
A
XE-991 3 µM
baseline
30 min
0
sec
5
B
NB Frequency
Theta Peak Power
Alpha Peak Power
Hz
0.08
0.06
0.04
0.02
0.00
****
ns
BL
30 min
A.U.
1.5
1.0
0.5
0.0
**
vehicle
XE-991 3 uM
C
Bethanechol 100 µM
baseline
5 min
60 min
0
sec
300
10 sec
D
NB%
% change from baseline
50
0
-50
-100
-150
*
5 min
60 min
E
NB duration
60
40
20
-20
Bethanechol 100 µM

Fig. S7

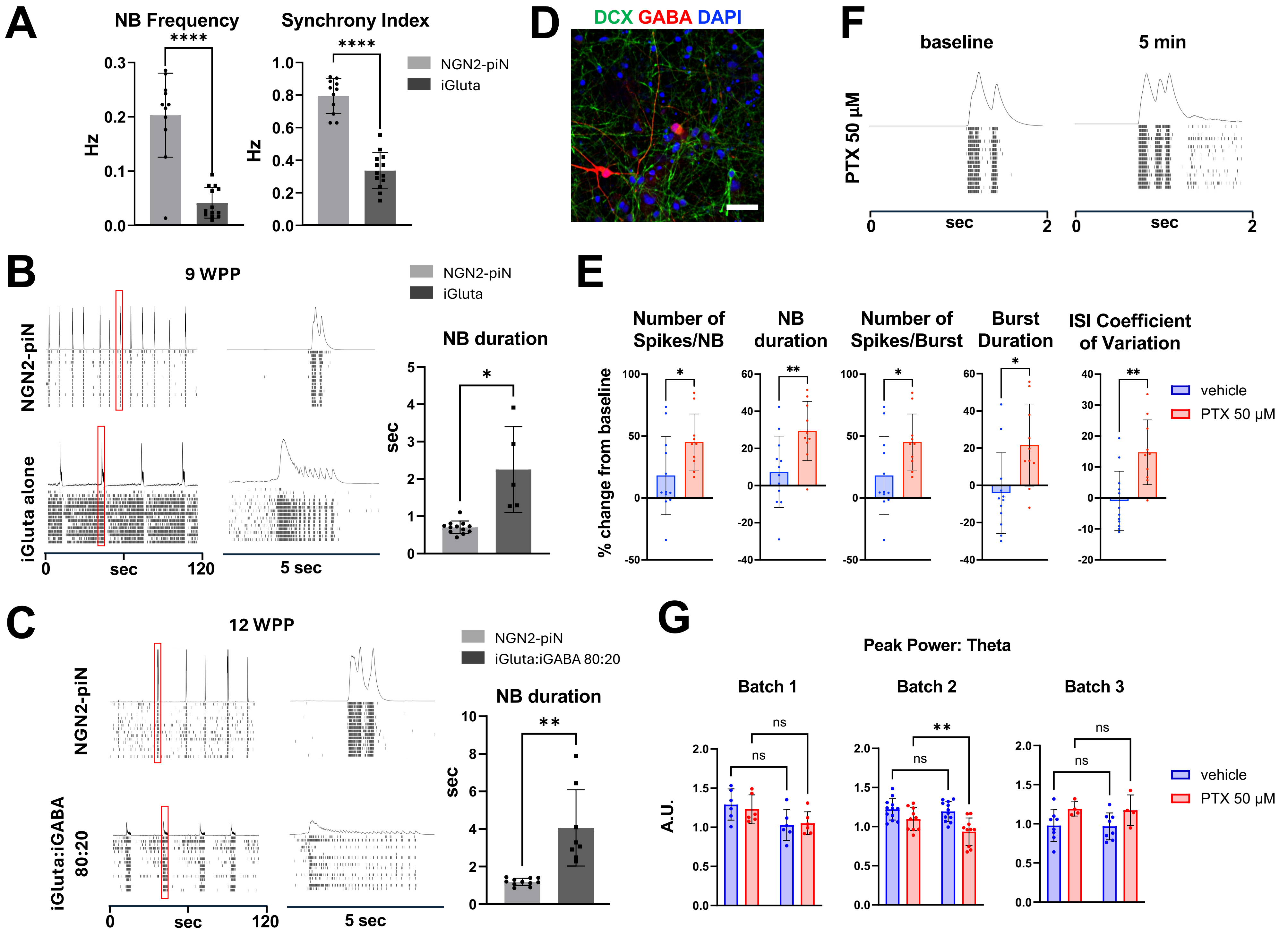
A
NB Frequency
Synchrony Index
Hz
NGN2-piN
iGluta
D
DCX GABA DAPI
F
baseline
5 min
PTX 50 µM
sec
B
9 WPP
NGN2-piN
iGluta alone
sec
120
5 sec
NB duration
E
Number of Spikes/NB
NB duration
Number of Spikes/Burst
Burst Duration
ISI Coefficient of Variation
% change from baseline
vehicle
PTX 50 µM
C
12 WPP
iGluta:iGABA 80:20
G
Peak Power: Theta
Batch 1
Batch 2
Batch 3
A.U.
ns
